# Genome-Wide Identification and Expression Analysis of the 14-3-3 (TFT) Gene Family in Tomato, and the Role of *SlTFT4* in Salt Stress

**DOI:** 10.3390/plants11243491

**Published:** 2022-12-13

**Authors:** Chunping Jia, Bin Guo, Baike Wang, Xin Li, Tao Yang, Ning Li, Juan Wang, Qinghui Yu

**Affiliations:** 1 Institute of Horticulture Crops, Xinjiang Academy of Agricultural Sciences (Key Laboratory of Genome Research and Genetic Improvement of Xinjiang Characteristic Fruits and Vegetables), Urumqi 830091, China; 2 College of Life Science and Technology, Xinjiang University, Urumqi 830046, China; 3 The State Key Laboratory of Genetic Improvement and Germplasm Innovation of Crop Resistance in Arid Desert Regions (Preparation), Urumqi 830091, China; 4 College of Computer and Information Engineering, Xinjiang Agricultural University, Urumqi 830052, China

**Keywords:** tomato, 14-3-3, TFT, genome-wide identification, evolutionary analyses, expression patterns, abiotic stresses, phytohormone treatments

## Abstract

The 14-3-3 proteins, which are ubiquitous and highly conserved in eukaryotic cells, play an essential role in various areas of plant growth, development, and physiological processes. The tomato is one of the most valuable vegetable crops on the planet. The main objective of the present study was to perform genome-wide identification and analysis of the tomato 14-3-3 (SlTFT) family to investigate its response to different abiotic stresses and phytohormone treatments in order to provide valuable information for variety improvement. Here, 13 SlTFTs were identified using bioinformatics methods. Characterization showed that they were categorized into ε and non-ε groups with five and eight members, accounting for 38.5% and 61.5%, respectively. All the SlTFTs were hydrophilic, and most of them did not contain transmembrane structural domains. Meanwhile, the phylogeny of the SlTFTs had a strong correlation with the gene structure, conserved domains, and motifs. The *SlTFTs* showed non-random chromosomal distribution, and the promoter region contained more *cis*-acting elements related to abiotic stress tolerance and phytohormone responses. The results of the evolutionary analysis showed that the *SlTFTs* underwent negative purifying selection during evolution. Transcriptional profiling and gene expression pattern analysis showed that the expression levels of the *SlTFTs* varied considerably in different tissues and periods, and they played a specific role under various abiotic stresses and phytohormone treatments. Meanwhile, the constructed protein-based interaction network systematically broadens our understanding of SlTFTs. Finally, the virus-induced gene silencing of *SlTFT4* affected the antioxidant and reactive oxygen species defense systems, increased the degree of cellular damage, and reduced salt resistance in tomatoes.

## 1. Introduction

The 14-3-3 proteins are part of the G-box protein complex, which is widespread in eukaryotes and highly conserved in structure, mainly in the form of homodimers or heterodimers; every monomer consists of nine α-helices arranged in an antiparallel fashion, and acts as a dimeric phosphoserine binding protein that can interact with two target proteins simultaneously or with two structural domains of one target protein. After binding to a phosphorylated serine/threonine residue of a small shared sequence on the target protein, it mainly interacts with its target protein through three phosphorylation binding modes to ensure its important regulatory function in the organism [1,2,3,4,5,6,7]. The members of the 14-3-3 gene family are classified into ε and non-ε groups based on their gene structure and amino acid (AA) sequence similarity. Typically, the former gene structure contains six to seven exons and four to six introns, while the latter generally contains only four exons and three introns [8,9,10].

The nomenclature of higher plant 14-3-3 in different species has not yet been standardized, with some being referred to as G-box factor 14-3-3 homologs (GF14s) or general regulatory factors (GRFs) [8,11]. In tomatoes, the nomenclature Tomato Fourteen-Three-three (TFTs) is commonly used [12]. The 14-3-3 proteins serve as a link between protein interactions. They activate target proteins by recognizing specifically phosphorylated or non-phosphorylated sequences and interacting with a wide range of target proteins, including protein kinases, transporter proteins, metabolic enzymes, transcription factors (TFs), and ion channels [13]. They also modify their subcellular localization, regulate gene transcription, and affect their structural stability, thus playing key roles in a wide range of physiological processes, such as plant growth and development, signal transduction, cellular metabolism, post-harvest maturation, response to adversity and nutrient stress, and plant–pathogen interactions [14,15].

For example, in *Arabidopsis thaliana*, At14-3-3μ/ν affect flowering transition and early photosensitive response [16]. In pears, *Pp14-3-3a* is modulated during fruit ripening senescence and participates in the fruit response to salicylic acid (SA) and ethylene (ET) signaling [17]. In bananas, most *14-3-3s* may be engaged in adjusting early fruit development, post-harvest ripening, and the response to abiotic stresses [18]. The overexpression of *AtGRF9*, *At14-3-3λ*, and *ZmGF14-6*/*OsGF14c* improved drought resistance in transgenic *A. thaliana*, cotton, and rice, respectively [19,20,21,22], while the silencing of *AtGF14m* improved drought resistance in *A. thaliana* at the seed germination and seedling stages [23]. In *A. thaliana*, At14-3-3λ/κ and AtRCI1A have been shown to negatively regulate salt tolerance and cold tolerance, respectively [20,24,25], and *AtGRF5*/*9*/*11* play vital roles in the regulation of metabolic pathways during nitrogen deficiency, phosphorus starvation, and iron deficiency responses, respectively [26,27,28]. In soybean, *SGF14c*/*l* play a critical role in the early developmental stage of root nodules [29]. In rice, *OsGF14b/c/d/e/f* were significantly up-regulated in expression under low temperature stress [30].

In recent years, more and more studies on the interaction of 14-3-3 proteins with other proteins have been conducted, and more than 300 target proteins interacting with 14-3-3 proteins have been appraised in plants with the help of affinity chromatography and yeast two-hybrid [31,32]. For example, in *A. thaliana*, At14-3-3ω and AtCDPK16 can affect ET synthesis in plants by regulating the stability of AtACS7, the rate-limiting enzyme of ET synthesis [33]. At14-3-3S interacts with the transcription factor AtWRI1, and their transient co-expression promotes the biosynthesis of vegetable oil in tobacco leaves. The seed oil content of transgenic plants overexpressing At14-3-3 was increased [34]. Yeast two-hybrid and immunoprecipitation analysis confirmed that At14-3-3χ interacts with ATL31, which targets and ubiquitinates the 14-3-3 protein for degradation through the ubiquitin-proteasome system and thus participates in plant responses to cellular carbon (C)/nitrogen (N) stress [35]. The yeast two-hybrid technique confirmed that the rice 14-3-3 protein can interact with 1-aminocyclopropane-1-carboxylic acid synthase, which is involved in ET synthesis [36]. In barley, the transcription factor HvABI5 of the abscisic acid (ABA)-responsive element binding factor protein family was found to bind to the *cis*-acting element of the ABA-inducible HVA1 promoter and to interact with three 14-3-3 proteins by yeast two-hybrid experiments, demonstrating that the 14-3-3 junctional protein is a key component of the seed intermediate in ABA signaling during germination [37]. 

In tomatoes, it has been shown that the 14-3-3 protein acts as an intracellular receptor for Hd3a florigenin, which affects the florigenin flowering pathway in tomatoes, and tomato productivity can be fine-tuned and optimized by using combinations of selected mutations in multiple florigens in pathway components [38]. Similarly, FTL1, a FLOWERING LOCUS T paralog, interacts with 14-3-3/2 to form a flowering activation complex, with SELF-PRUNING-interacting G-BOX, regulating tomato flowering [39]. During blue light-induced photomorphogenesis, the expression of *SlTFT9*, but not *SlTFT6*, is regulated by phytohormone cytokinins. 

Many studies have found that multiple abiotic stresses alter the transcriptional and protein expression levels of *SlTFTs*, suggesting that *SlTFT* transcription and translation can respond to abiotic stresses [15,31,40,41]. For example, *SlTFT7* may mediate the interactions between the signal transduction pathways in response to salt stress, potassium deficiency, and iron deficiency in tomatoes [12]. Arbuscular mycorrhizal (AM) symbiosis may regulate stomatal behavior and maintain water use efficiency (WUE) by regulating the *14-3-3* gene in the ABA signaling pathway, thereby improving drought tolerance in tomatoes [42]. The growth of transgenic *A. thaliana* overexpressing *SlTFT4* was significantly enhanced under alkaline stress compared with the wild type (WT), and *SlTFT4* acts as a regulator of H^+^ efflux, basal IAA transport, and PKS5-J3 pathway integration in the root alkaline stress response and coordinates the root tip response to alkaline stress to maintain primary root elongation [43]. Transgenic *A. thaliana* overexpressing *SlTFT6* and *SlTFT7* both showed a great promotion of growth under low phosphorus (LP) stress compared to the WT. The former showed reduced starch synthase activity, reduced starch content, and increased sucrose loading in the phloem under LP stress. In the latter, root tip H^+^ flux and root plasma membrane (PM) H^+^-ATPase activity were significantly increased under LP stress. *SlTFT6* acts mainly on leaves to promote root growth by regulating leaf carbon allocation and increasing sucrose transport in the phloem and is involved in the systemic response to LP, while *SlTFT7* acts directly on roots by activating the root PM H^+^-ATPase, which releases more protons under LP [44]. 

Meanwhile, a growing body of evidence supports the prominent role of SlTFTs in regulating tomato immunity to various pathogens. Many studies have shown that SlTFTs can alter their transcript levels or properties in response to pathogen infection [15,31,40,41]. The *SlTFTs* were down-regulated in the interaction of resistant tomatoes with *Verticillium dahliae*. However, *SlTFT1*/*4*/*6* were induced after treatment with a fungal toxin, Fusicoccin [40]. The silencing of *SlTFT7* in leaves did not affect tomato susceptibility to *Macrosiphum euphorbiae*, but it increased the longevity and fertility of *Aphis gossypii* [45]. XopN interacts with SlTFT1, which functions in PAMP-triggered immunity and is a virulence target of XopN. The silencing of *SlTFT1* in leaves resulted in the increased growth of *Xanthomonas campestris* pv *vesicatoria*, indicating that SlTFT1 is required to suppress *Xcv* proliferation. The induction of *PTI5*, *GRAS4*, *WRKY28*, and *LRR22* mRNAs by *Xcv* requires the expression of *SlTFT1* [46]. The *Hrp outer protein Q* effector from the *Pseudomonas syringae* pv *tomato* strain DC3000 binds to a variety of tomato 14-3-3 proteins, including SlTFT1 and SlTFT5. 

Currently, with the continuous release of the genome sequences of different species, the genome-wide identification and analysis of the *14-3-3* gene family has been completed within several species, including model crops (*A. thaliana* and rice) [8,9,11,30], grain crops (wheat and millet) [47,48], cash crops (soybean and cotton) [49,50], fruit crops (banana, grape, apple, and mango) [18,51,52,53], and other plants (poplar, rubber tree, and alfalfa) [10,54,55].

The tomato is an important vegetable crop and a model plant of the Solanaceae family. The cloning and the identification of *SlTFTs* have also attracted the attention of researchers. They appear to play a regulatory role in abiotic stresses (such as drought, salt, alkali, and low phosphorus) and biotic stresses (such as *Xanthomonas euvesicatoria* and *M. euphorbiae*) [12,42,43,44,45,56]. Nevertheless, the functions of the majority members of the *SlTFT* family remain unclear. With the rapid development of bioinformatics and modern molecular biology technical approaches, it is necessary to further analyze and refine the properties and functions of the entire family.

Presently, we conducted the genome-wide identification of *SlTFTs* and the systematic analysis of their phylogeny, gene structure, conserved domains, motifs, phylogenetic trees among different species, chromosome distribution, purification pressure, replication events, synteny and divergence time among different species, *cis*-acting elements, transcriptional profiles, expression patterns under various abiotic stresses and phytohormone treatments, VIGS, and protein interaction networks. The main purpose of the present study is to provide new and comprehensive information on the *SlTFT* family and to provide a reference value for gene molecular and biological functions and genetic breeding studies.

## 2. Results

### 2.1. Identification and Characterization of SlTFTs

In the present study, a total of 13 TFTs, named SlTFT1–SlTFT13, were identified from the tomato genome, of which SlTFT13 is a newly identified SlTFT. Meanwhile, the genomic, the coding region (CDS), and the protein sequence information of all the SlTFTs are shown in Supplementary Table S1. The SlTFTs as a whole could be divided into ε and non-ε groups, with five and eight members, accounting for 38.5% and 61.5%, respectively. Their AA lengths ranged from 243 AA (SlTFT7) to 401 AA (SlTFT2), with an average of 278.9 AA. The molecular weight (MW) ranged from 27.76 kDa (SlTFT7) to 45.15 kDa (SlTFT72), and the isoelectric point (pI) ranged from 4.61 (SlTFT8) to 5.4 (SlTFT3), all of which were acidic proteins (pI < 7). The instability coefficients ranged from 39.24 (SlTFT6) to 54.17 (SlTFT2), and all the SlTFTs except SlTFT1 and SlTFT6 had low instability indices (<40), indicating that 84.6% of the SlTFTs were unstable at the theoretical level (Table 1). The grand average of the hydropathy (GRAVY) index indicated that all the SlTFTs were hydrophilic (GRAVY < 0). The predicted possible subcellular localization of all the SlTFTs revealed that all members were localized in the cytoplasm, except SlTFT7, which is presumably beneficial for strengthening the interaction between the nucleus and the cell membrane, thus establishing a protective mechanism to effectively respond to various external stressful environments (Table 1). 

**Figure 1:**
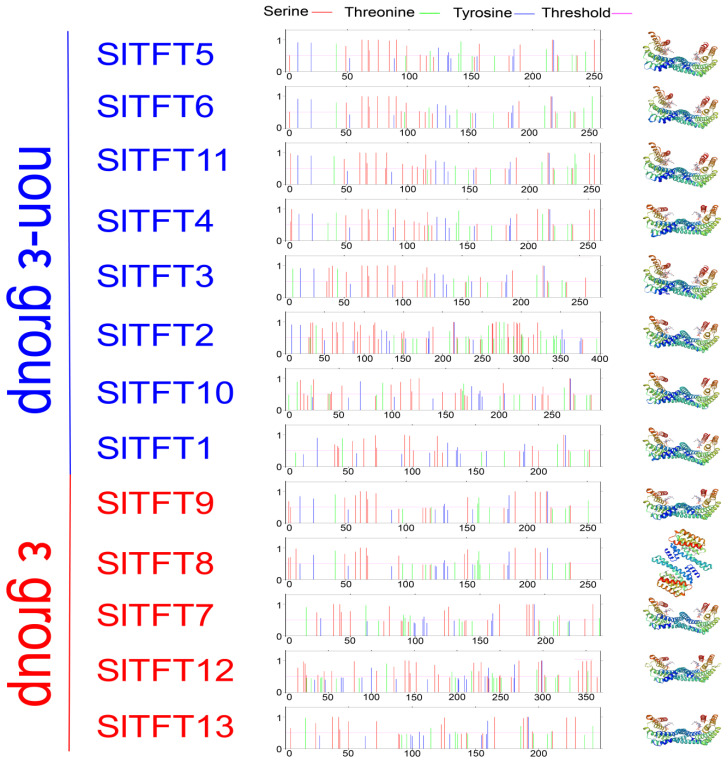
The predicted phosphorylation sites and protein tertiary structure prediction of SlTFTs. The vertical red, green, and blue lines in the middle graph represent the predicted phosphorylation site scores for serine, threonine, and tyrosine, respectively. The horizontal pink line represents the average of the predicted score threshold of 0.5. The gradient colors in the tertiary structure of the protein on the right indicate amino acids from the N-terminal to the C-terminal, different helices, and folded regions with color transitions from blue to red colors.

The model with the largest global model quality estimate (GMQE) score (between 0 and 1), i.e., the most reliable model, was selected as the tertiary structure model for the SlTFTs. They are mainly formed by secondary structures such as α-helix, β-folding, β-turning, and irregular coiling by further coiling and folding through the interaction of side chain groups with the help of secondary bond maintenance. Apart from SlTFT8, the remaining members of the SlTFTs have the same or similar typical groove structures, suggesting that they may perform similar functions. The NetPhos 3.1 server uses neural network integration to forecast the Ser, Thr, or tyrosine (Tyr) phosphorylation locations in eukaryotic proteins. We found that SlTFTs generally have multiple different phosphorylation sites on their AA sequences, and the results are generally scored (0–1) with a multiple above 0.5, which shows that the results are positive, indicating that general and kinase specificity is also predicted. It is hypothesized that the proteins with different structures and kinase-specific phosphorylation sites determine the functional diversity of the SlTFTs (Figure 1). The Tied Mixture Hidden Markov Model (TMHMM) is a program for predicting transmembrane helices based on the Hidden Markov Model (HMM) model, which integrates the properties of the hydrophobicity, charge bias, helix length, and topological constraints of the membrane proteins in the transmembrane region and the inner and outer membrane regions as a whole. Because it is particularly good at distinguishing soluble proteins from membrane proteins, it is often used to determine whether a protein is a membrane protein. Our prediction results show that all the SlTFTs have no transmembrane helices, except for SlTFT12 which predicts two transmembrane helices, implying that the majority of them are membrane proteins that do not contain transmembrane structural domains (Supplementary Table S2).

### 2.2. Phylogeny, Gene Structure, Conserved Domains, and Motif Analysis of SlTFTs

**Figure 2:**
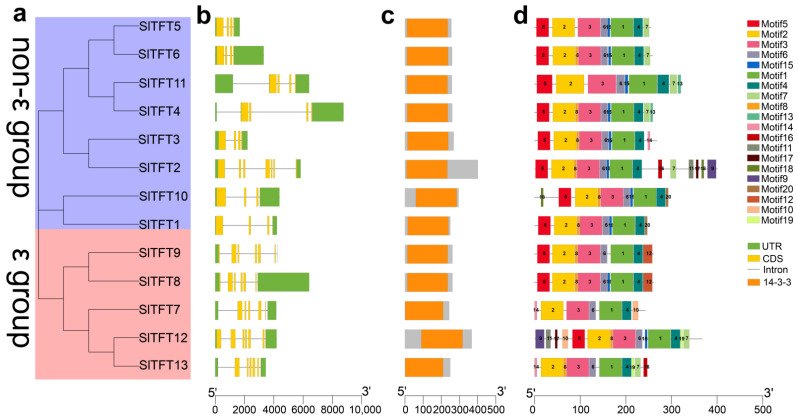
Phylogeny, gene structure, conserved domains, and motif of SlTFTs. (**a**) Phylogenetic tree of SlTFTs. The blue-purple area represents clustering to the 8 members of the non-ε group, and the orange area represents clustering to the 5 members of the ε group. (**b**) Structure of exons, introns, and untranslated regions (UTR) in *SlTFTs*. The green box represents the UTR region in the gene structure, the yellow box represents the CDS region in the gene structure, and the thin gray line represents the intron region in the gene structure. (**c**) Distribution of conserved domains of SlTFTs. The gray boxes represent protein regions composed of AA, and the orange boxes indicate that they contain 14-3-3 conserved domains. (**d**) Different motifs contained in SlTFTs. Each of the 20 different gradient colors represents 20 different motifs.

To investigate the homology and similarity of the identified SlTFTs, an unrooted phylogenetic tree was constructed according to the alignment of all the SlTFT protein sequences. Phylogenetic analysis indicated that they could be categorized into two groups belonging to three major branches, i.e., the first two major branches constituted the non-ε group, while the last major branch constituted the ε group (Figure 2a). Meanwhile, the phylogeny of the SlTFTs had a strong correlation with the gene structure, conserved domains, and motifs. Gene structure analysis revealed that the *SlTFTs* have different exons, introns, and structural features. We found that the *SlTFTs* contained four to seven exons and three to six introns. The non-ε groups, except for SlTFT2, all contained only four exons and three to four introns. In contrast, the members of the ε group had five to approximately seven exons and six introns (Figure 2b). It is hypothesized that there is intron loss and additional exon gain for genes in the tomato ε group and non-ε group, and that this exon–intron structural diversity also indicates differences in the amplification and evolution of the genes in the different groups. Conserved domain analysis revealed that members of the SlTFTs subgroup all contain only the same protein domain, implying that their primary functions may be consistent (Figure 2c). Our further conserved motif analysis showed that the motif composition of all the SlTFT subgroup members was relatively similar except for SlTFT2 and SlTFT12 (Figure 2d). Each SlTFT subgroup member contains several identical motifs specific to the group, and this taxon specificity implies that the same taxon has similar biological functions. Motifs 1, 2, 3, 4, and 6 are present in all SlTFTs and are highly conserved, suggesting that they may be required for protein function. Moreover, the formation of motif patterns suggests that SlTFTs are actively involved in various biological processes.

### 2.3. Phylogenetic Tree Analysis of 14-3-3 Proteins among Different Model Species

**Figure 3:**
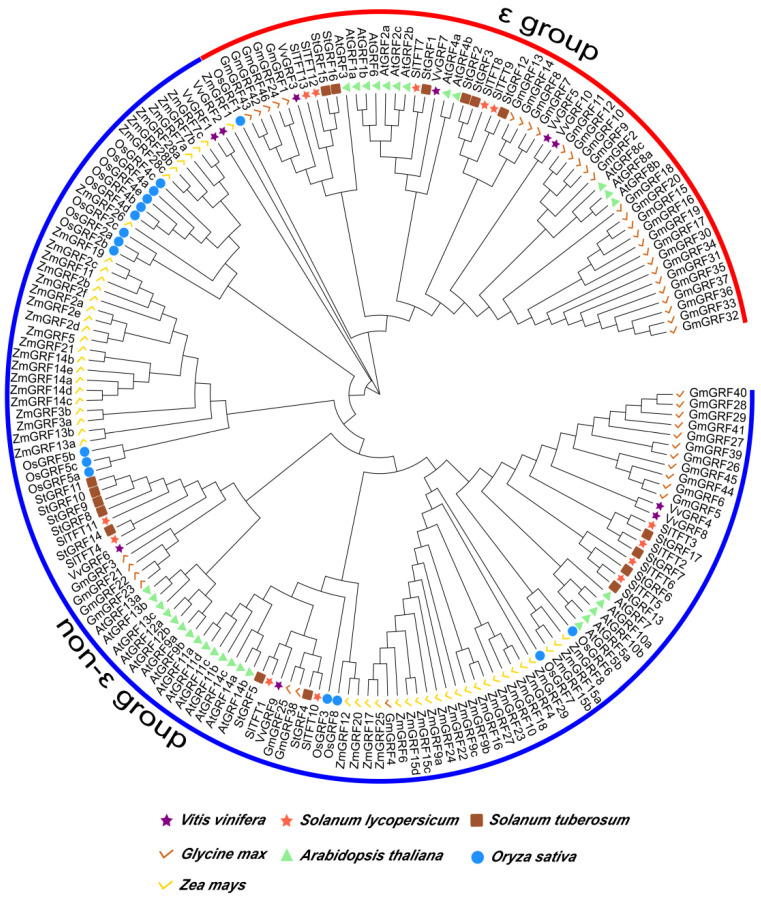
Phylogenetic analysis among SlTFTs and six other species. All 170 14-3-3s were compared in multiple sequences and a phylogenetic tree was constructed using the maximum likelihood method and 1000 bootstrap resamples in MEGA 11 software. The different color shapes represent the 14-3-3s in different species. Red and blue colors in the outer circle represent different groups, respectively.

To better understand the evolutionary relationships of the SlTFT family members among different species, we used the ML method to construct a phylogenetic tree of the SlTFT family using 14-3-3 protein sequences from two monocots (rice and maize) and four dicots (*A. thaliana*, grape, potato, and soybean) species (Figure 3). Based on the phylogenetic relationships, the 170 14-3-3 proteins were divided into two main groups: the ε group and the non-ε group, each consisting of 56 and 114 members. The members in the common group imply that they have comparable origin and evolutionary relationships. The different groups all contain members of 14-3-3s from multiple species, and their origins presumably appeared before the divergence of monocotyledons and dicotyledons. The SlTFTs and StGRFs were mostly clustered next to each other in a branch, indicating that their AA sequence similarity was closer. Some subbranches contain members of only one species, while others contain members of multiple species; presumably, the 14-3-3s are more readily divergent in structure and function, suggesting a diversity of the gene expression and function of the family members.

### 2.4. Chromosome Distribution, Replication Events, Divergence Time, and Synteny Analysis of SlTFTs

**Figure 4:**
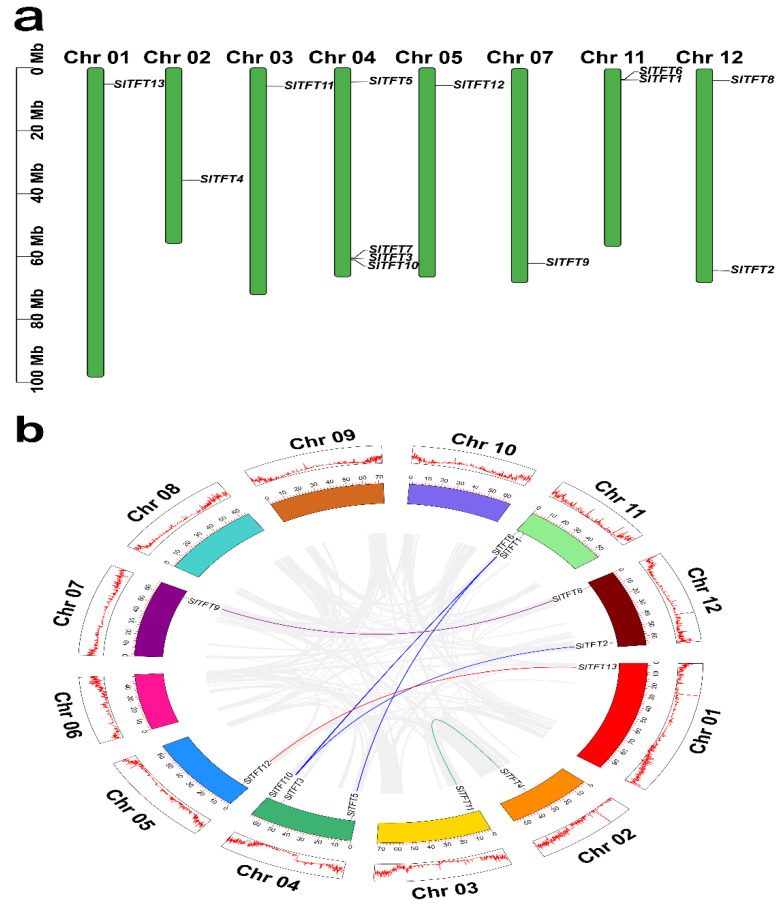
Chromosomal localization and gene duplication events in *SlTFTs*. (**a**) Distribution of genes on tomato chromosomes. The green rectangular squares represent chromosomes with specific chromosome numbers and the location and number of *SlTFTs* distributed on the chromosomes, with a scale of chromosome lengths on the left. (**b**) Duplicate pairs of *SlTFTs* in tomato. From outside to inside, the first circle is the chromosome number and density, and the second circle is the ideogram of the chromosome, with the scale on top indicating the coordinate position of the chromosome. Different colored lines indicate synteny pairs of *SlTFTs*, while gray lines indicate synteny pairs of all tomato genes.

The genomic distribution of *SlTFTs* varies depending on the chromosome; they are distributed on the remaining eight chromosomes except chromosomes 6, 8, 9, and 10, with one member on each of the chromosomes 1, 2, 3, 5, and 7, two members on each of the chromosomes 11 and 12, and four members on chromosome 4. Almost all the SlTFTs are distributed at both ends of the chromosome (Figure 4a). The results of the intra-genomic replication events analysis of all the *SlTFTs* showed that we did not find tandem replication gene pairs, while 12 *SlTFTs* were found to exhibit seven pairs of segmental duplication events scattered on the corresponding chromosomes (Figure 4b). It may be that *SlTFTs* belonging to the same group in the phylogenetic tree are not localized on the same chromosome; rather, they are arranged in different positions on the same chromosome or scattered on different chromosomes. It is implied that segmental duplication rather than tandem replication plays a critical role in the amplification of the *SlTFT* family.

Our assessment of species selection and evolution showed that the nonsynonymous substitutions (*K*a)/synonymous substitutions (*K*s) ratios of the segmental duplication events in the *SlTFTs* fell in the range of 0.04 to 0.14, with a mean value of 0.09. As the ratios of all the segmental duplication gene pairs were much less than 1, this suggests that *SlTFTs* have undergone a strong purifying selection during evolution. Then, we used the *K*s values to predict the divergence time of the duplication events in *SlTFTs*. Segmental duplication in *SlTFTs* occurred between 15.61 and 54.19 million years ago (MYA), with a mean value of 31.51 MYA (Supplementary Table S3).

The divergence time is a current hot topic in macroevolutionary analysis. Usually, the genetic distance between two species is proportional to the divergence time of the species. Synteny analysis is an essential analytical strategy in comparative genomics. This is because it allows the analysis of molecular evolutionary events between species at large scales (estimates of rearrangement and duplication events within the genome) and small scales (for base substitution rates as well as insertion and deletion events at the genome level). In summary, it is important to investigate the divergence time and synteny analysis in species.

**Figure 5:**
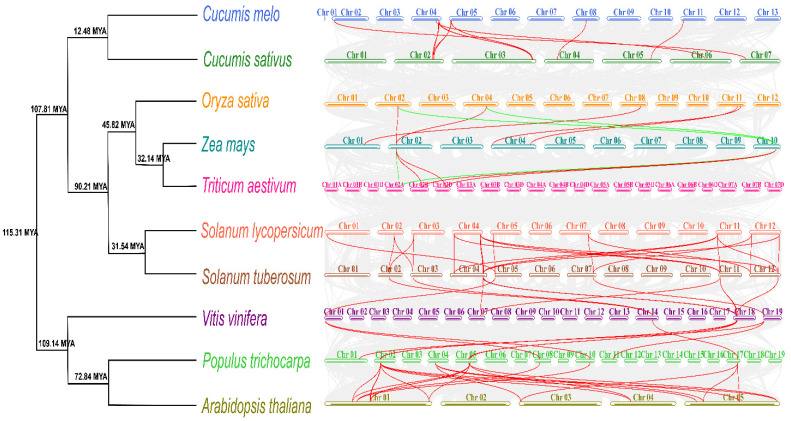
Divergence times and synteny of *14-3-3s* among the 10 species. The left side of the figure shows the constructed evolutionary divergence tree with the divergence time labeled above the branches. In the middle are the Latin names of 10 different species. The long bars of different colors on the right side represent the chromosomes of different species. The gray line indicates the synteny of all genes between different species. The red line indicates the synteny of *14-3-3s* between different species. The green line indicates the synteny between the *14-3-3s* of one species and the non-*14-3-3s* of another species.

By constructing the divergence time trees and synteny profiles among the *SlTFTs*, three monocotyledons (rice, maize, and wheat), and six dicotyledons (watermelon, melon potato, grape, poplar, and *A. thaliana*), we further understood the divergence time and synteny relationships of the *SlTFTs* with other species and *14-3-3s* among other species (Figure 5). The results showed that the 10 species were mainly divided into two evolutionary branches at around 108 MYA, with tomato and potato having similar divergence times with rice and maize at around 32 MYA, while the divergence times with other species were farther apart. In monocotyledonous plants of rice, wheat, and maize, in addition to the synteny between the *14-3-3s* (shown by red lines), there is also synteny between the homologous genes (shown by green lines). More synteny relationships between each of the *14-3-3s* exist in dicotyledonous plants. Some *14-3-3s* form two to four synteny gene pairs, and they may have played an essential role in the evolution of the *14-3-3* family. Between tomato and potato, *14-3-3s* have the most synteny relationships, probably because they belong to the same family of Solanaceae. Notably, no synteny relationships of *14-3-3s* were found between tomato and wheat, rice, and maize, and between melon and rice, suggesting that there may not be synteny relationships of *14-3-3s* between monocotyledons and dicotyledons, indirectly indicating that there is variability and diversity in the structure and function of *14-3-3s* (Supplementary Figure S1).

### 2.5. Analysis of Cis-Acting Elements of SlTFTs

*Cis*-acting elements are found in sequences next to genes that can affect gene expression, and they include core promoters, enhancers, and silencers, whose role is to participate in the regulation of gene expression. The *cis*-acting element itself does not encode any protein but only provides an action site to act by interacting with the *trans*-acting factor. The prediction of *cis*-acting elements and the identification and characterization of *cis*-regulatory sequences and their functional studies in co-regulating development and accomplishing environmental responses are essential for botany.

**Figure 6:**
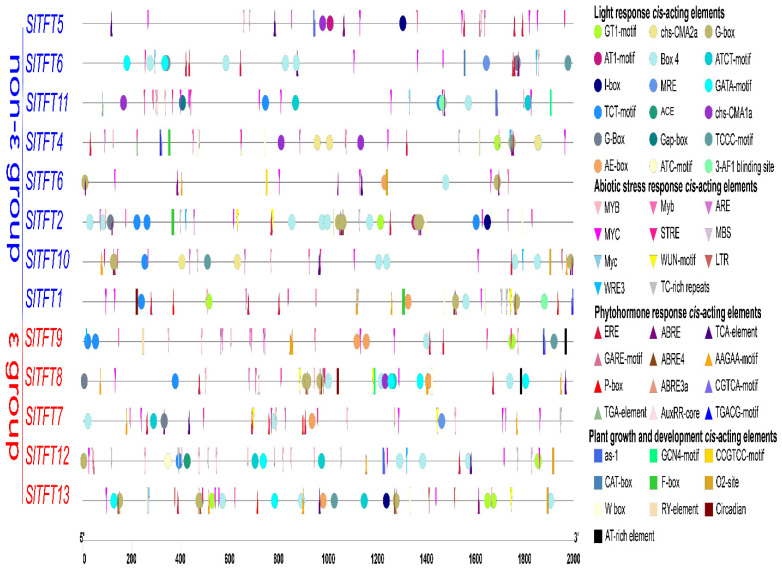
Predicted *cis*-acting elements in the promoter regions of *SlTFTs*. Gene names are arranged according to the order of the phylogenetic tree. The different color fonts on the left side are the grouping of *SlTFTs* and the corresponding members. The thin gray line in the middle represents sequences within 2000 bp upstream of the initiation codon of each *SlTFTs*, and the scale of base lengths at the bottom, where different colors and shapes of patterns represent different classes of *cis*-acting elements. A detailed division of the four major categories of *cis*-acting elements is provided on the right.

We discovered that in addition to the common *cis*-acting elements, TATA box and CAAT box, in the promoter regions of *SlTFTs*, the other *cis*-acting elements present can be divided into four categories (Figure 6). The first category comprises the light-responsive *cis*-acting elements. All *SlTFTs* contain 18 such *cis*-acting elements, including Box4, GT1-motif, chs-CMA2a, and G-box, and all the promoter regions of *SlTFTs* contain at least three or more light-responsive *cis*-acting elements. The second category comprises the *cis*-acting elements associated with responses to abiotic stress environments or external stresses. Eleven such *cis*-acting elements, including MYB, MYC, and LTR, suggest that *SlTFTs* may play a key role in processes such as drought, salt, or low-temperature abiotic stress defense and stress. The third category comprises the phytohormone response *cis*-acting elements, including 12 such *cis*-acting elements, such as ABRE, TGACG-motif, and P-box, which are associated with the response of different hormones, such as ABA, methyl jasmonate (MeJA), and gibberellin (GA), respectively. The fourth group is involved in the plant growth and development of *cis*-acting elements, such as GCN4 motif, CAT box, and circadian, and seven such *cis*-acting elements are associated with growth and developmental processes, such as the endosperm expression, meristem expression, and circadian rhythm in tomato. In addition, some *SlTFTs* also contain F-box with substrate recognition properties during ubiquitin-mediated protein hydrolysis and W-box with binding sites for WRKY TFs.

### 2.6. Transcription Profiling of SlTFTs

**Figure 7:**
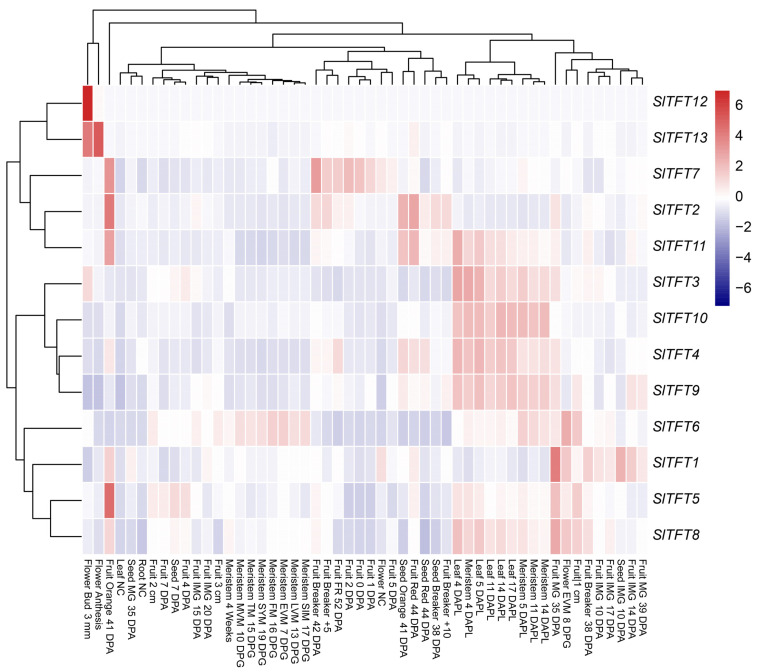
Clustering heat map of the expression patterns of *SlTFTs* in various tissues. The top of the figure indicates the hierarchical clustering results, and the bottom indicates the seeds, roots, meristematic tissues, leaves, flowers, and fruits at different periods in cultivated tomatoes. The left side of the horizontal coordinate shows the hierarchical clustering results, and the right side shows the expression patterns of all 13 *SlTFTs*. The gradient from blue to red indicates the expression from low to high.

To explore the potential functions of *SlTFTs*, the transcript profile expression data for each *SlTFT* were systematically analyzed. The expression modes of the *SlTFTs* in the various tissues were presented as clustered heat maps (Figure 7). We found that *SlTFTs* were expressed in the multiple tissues examined and that the expression levels varied greatly among the tissues and periods. *SlTFTs* generally have low expression in seeds, flowers, and roots and generally have high expression in leaves, meristematic tissues, and fruits. Among them, certain genes exhibited unique expression profiles. For example, *SlTFT1*/*2*/*4*/*5*/*7*/*8*/*11* are highly expressed in tomato fruits and possibly participated in the growth and development process from immaturity to maturity. *SlTFT3*/*4*/*6*/*8*/*9*/*10*/*11* are highly expressed in leaves (4/5/11/14/17 DAPL) and meristematic tissues (4/5/11/14 DAPL) and may play a regulatory effect in these periods. In addition, *SlTFT3*/*12*/*13* and *SlTFT13* are highly expressed in the flower bud (3 mm) and flowering stage, respectively, and may play a regulatory role in the early and full periods of flower development. Among them, *SlTFT4*/*8*/*11* are highly expressed in leaves, meristematic tissues, and fruits; *SlTFT3* was highly expressed in leaves, meristematic tissues, and flower buds; and *SlTFT13* is highly expressed in flower buds and anthesis, and they possibly closely participated in these growth and developmental processes. The above analysis of the expression patterns of *SlTFTs* provides additional insight into their possible roles.

### 2.7. Analysis of Expression Patterns of SlTFTs in Response to Abiotic Stresses and Phytohormone Treatments

**Figure 8:**
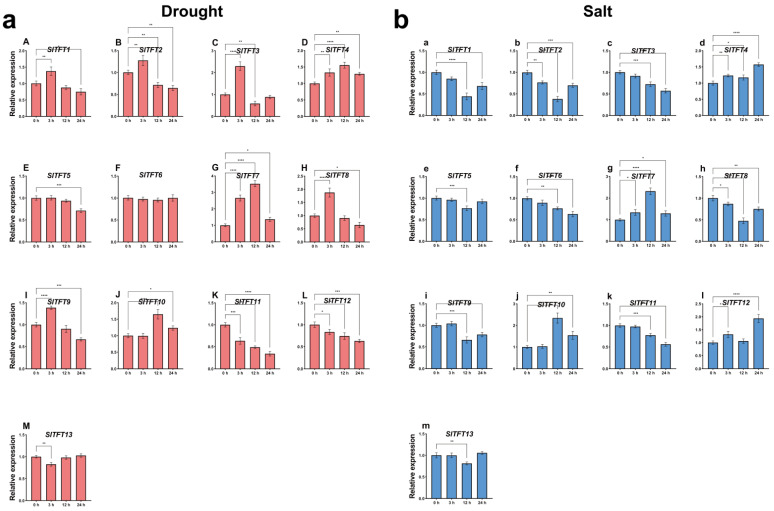
Responses of 13 *SlTFTs* to different abiotic stress salt (**a**) or drought (**b**) treatments. (**a**) A to M represent *SlTFT1* to *SlTFT13*, respectively. The horizontal coordinates in the figure are the four different treatment periods under 200 mM NaCl treatment, and the vertical coordinates are the relative expression of each *SlTFT* gene. (**b**) a to m represent *SlTFT1* to *SlTFT13*, respectively. The horizontal coordinates in the figure are the four different treatment periods under 300 mM D-mannitol treatment, and the vertical coordinates are the relative expression of each *SlTFT* gene. Error bars are the standard deviations (SD) of three independent biological replicates, presented as mean ± SD. The statistical significance of differences was confirmed by Dunnett’s multiple comparison test (* *p* < 0.05, ** *p* < 0.01, *** *p* < 0.001, and **** *p* < 0.0001).

We also investigated the general expression characteristics of *SlTFTs* in response to different abiotic stresses and phytohormone treatments to better understand the possible roles of various *SlTFT* family members. We observed that under regular circumstances, no significant differences occurred in the expression changes of all the *SlTFTs* at all the periods under the control treatment (Supplementary Figure S2). However, under simulated high salt stress, the expression of most of the *SlTFTs* was significantly down-regulated, whereas the expression of *SlTFT4*/*7*/*10*/*12* was significantly up-regulated. *SlTFT4*/*7* remained significantly highly expressed during all periods. *SlTFT7*/*10* showed a highly significant increase in expression of about 2.5-fold at the mid-stage (12 h) and the *SlTFT12* expression increased extremely significantly by about 2-fold at the later stage (24 h) (Figure 8a). Under simulated drought stress, the expression of *SlTFT11*/*12* continued to be significantly down-regulated throughout all periods, and the expression of *SlTFT5*/*13* was significantly down-regulated at the initial stage (3 h) and at 24 h, respectively. The expression of *SlTFT6* did not alter significantly over the entire period. The expression of *SlTFT4*/*7* continued to be significantly up-regulated throughout the entire period, with *SlTFT7* at 12 h showing a highly significant increase in expression of more than 3-fold. The expression of *SlTFT10* was significantly up-regulated at 12 h and significantly down-regulated at 24 h. The expression of *SlTFT1*/*2*/*3*/*8*/*9* was significantly up-regulated at 3 h and significantly down-regulated at 12 or 24 h (Figure 8b).

**Figure 9:**
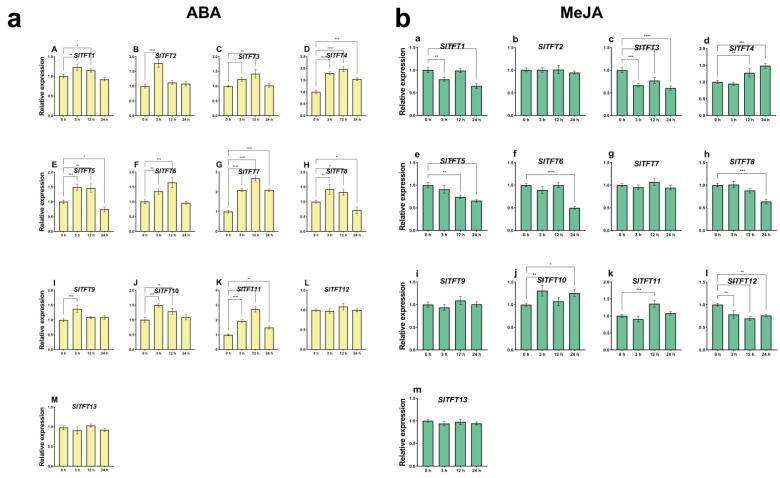
Responses of 13 *SlTFTs* to different phytohormone ABA (**a**) or MeJA (**b**) treatments. (**a**) A to M represent *SlTFT1* to *SlTFT13*, respectively. The horizontal coordinates in the figure are the four different treatment periods under 100 µM ABA treatment, and the vertical coordinates are the relative expression of each *SlTFT* gene. (**b**) a to m represent *SlTFT1* to *SlTFT13*, respectively. The horizontal coordinates in the figure are the four different treatment periods under 100 µM MeJA treatment, and the vertical coordinates are the relative expression of each *SlTFT* gene. Error bars are the standard SD of three independent biological replicates, presented as mean ± SD. The statistical significance of differences was confirmed by Dunnett’s multiple comparison test (* *p* < 0.05, ** *p* < 0.01, *** *p* < 0.001, and **** *p* < 0.0001).

Similarly, under the control treatment of double-distilled water (ddH_2_O) spraying, no significant difference in expression changes occurred for all the *SlTFTs* over the full time period (Supplementary Figure S3). However, under the phytohormone ABA treatment, the expression of *SlTFT4*/*7*/*11* continued to be significantly up-regulated throughout all the periods, with peak expression at 12 h. The expression of *SlTFT5*/*8* was significantly up-regulated at 3 h and 12 h, and significantly down-regulated at 24 h. The expression of *SlTFT2*/*9* was significantly up-regulated at 3 h. The expression of *SlTFT1*/*2*/*6*/*10* was significantly up-regulated at 3 h and 12 h. No significant difference in the expression changes of *SlTFT12*/*13* occurred in all the periods (Figure 9a). Under the phytohormone MeJA treatment, the expression of *SlTFT3*/*12* continued to be significantly down-regulated for the entire period. The expression of *SlTFT1*/*5*/*6*/*8* was significantly down-regulated in some periods. The expression of *SlTFT4* was significantly up-regulated at 12 h and 24 h. The expression of *SlTFT11* was significantly up-regulated at 12 h. The expression of *SlTFT10* was significantly up-regulated at 3 h and 24 h. The expression of *SlTFT2*/*7*/*9*/*13* did not change significantly in all the periods (Figure 9b).

The different expression patterns of these *SlTFTs* described above suggest that they have specific roles under different stresses and phytohormone treatments. Overall, the expression of *SlTFTs* was more sensitive to drought stress and ABA treatment. We found that some *SlTFTs* showed similar expression profiles in response to different abiotic stresses, with *SlTFT4*/*7*/*10* being up-regulated in response to both high salt and drought stresses. Similarly, *SlTFT4*/*10*/*11* showed a similar up-regulated expression pattern in response to different phytohormone treatments. *SlTFT4*/*10* can respond to all of these treatment conditions simultaneously, and they may be key genes in the *SlTFT* family involved in defense and the response to external stresses.

### 2.8. Correlation between Cis-Acting Elements and Expression Patterns under Different Abiotic Stresses or Phytohormone Treatments

As mentioned above, certain *SlTFTs* exhibit similar up-regulated expression patterns in response to different abiotic stresses and phytohormone treatments, and it is necessary to further explore their regulatory mechanisms. We analyzed the correlation between the number of *c*is-acting elements involved in defense, the response to external stress, and the phytohormone response possessed by *SlTFT4/7/10/11* and the different expression patterns under abiotic stresses or phytohormone treatments (Supplementary Table S4).

The numbers of *cis*-acting elements (MYB, Myb, MYC, and MBS) possessed by *SlTFT4/7/10/11* in response to salt and drought were 12, 11, 9, and 7, respectively, which were closely related to the significant changes in their expression patterns under salt and drought stresses. Notably, *SlTFT4/7/10* showed an up-regulated expression pattern, whereas *SlTFT11* showed a down-regulated expression pattern, suggesting that it may be involved in negative regulation. In addition, *SlTFT4/7/10/11* possessed one, five, four, and two STRE elements, respectively, presumably in response to high-temperature stress, and *SlTFT10* also possessed an LTR element, which may also respond to low-temperature stress. The numbers of *cis*-acting elements (ABRE and AAGAA-motif) possessed by *SlTFT4/10/11* in response to ABA were two, one, and five, respectively, which was closely related to the significant change in their expression patterns under ABA treatment. Meanwhile, *SlTFT4/11* had two *cis*-acting elements (TGACG-motif and CGTCA-motif) in response to MeJA, respectively, which were closely associated with their significant changes in expression patterns under MeJA treatment. *SlTFT7/10* both had no *cis*-acting elements in response to MeJA, but *SlTFT10* changed significantly under MeJA treatment at 3 h and 24 h, which may be related to the presence of the phytohormone-responsive element ERE. In addition, *SlTFT4/11* had one *cis*-acting element (TGA-element) in response to auxin (IAA), *SlTFT7/10* had one *cis*-acting element (TCA-element) in response to SA, and *SlTFT10* had two *cis*-acting elements (P-box) in response to GA, respectively; presumably, they may respond to the above phytohormone treatments.

### 2.9. Interaction Network Analysis of 14-3-3 Proteins

**Figure 10:**
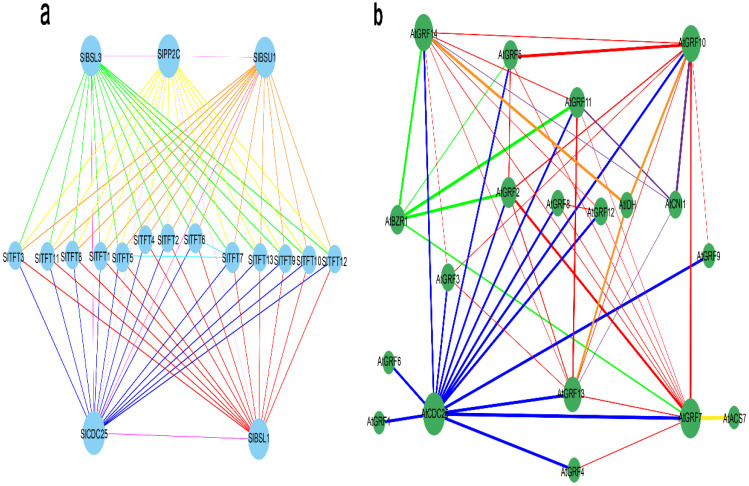
Protein interaction network diagram. (**a**) Protein interaction network between SlTFTs and other proteins in tomato. (**b**) Protein interaction network between AtGRFs and other proteins in *A. thaliana*. Lines of different colors and thicknesses represent the interactions between different proteins. Network construction was performed using String 11.5. In the basic settings, select the full STRING network (the edges indicate both functional and physical protein associations) as the network type. The line color indicates the type of interaction evidence chosen to indicate the meaning of network edges. Active interaction sources include text mining, experiments, databases, co-expression, neighborhood, gene fusion, and co-occurrence. The minimum required interaction score is medium confidence (0.40). The network display mode in the advanced settings is set to interactive SVG (network is a scalable vector graphic [SVG]; interactive). In the network display option, click the “hide disconnected nodes in the network” button.

Based on String 11.5 and Cytoscape 3.9.1, we constructed the interaction network between the SlTFTs and the other proteins. We found that all 13 SlTFTs interacted with five (SlBSL3, SlPP2C, SlBSU1, SlCDC25, and SlBSL1) other proteins, belonging to dual-specificity tyrosine-phosphatase, serine-/threonine-protein phosphatase, and protein kinase A-like kinase. Among the SlTFTs, SlTFT7 interacted with SlTFT5 and SlTFT6, respectively. Among the five other proteins, SlBSL1, SlBSL3, and SlBSU1 interacted with SlPP2C and SlCDC25, respectively, and SlPP2C and SlCDC25 interacted with each other and with all the other proteins (Figure 10a). As the model plant *A. thaliana* was more intensively studied, we also constructed the interaction networks between the AtGRFs and the other proteins, and we observed that all 14 AtGRFs were associated with five (AtBZR1, AtIDH-III, AtCNI1, AtCDC25, and ATACS7) other proteins belonging to the brassinosteroid signaling positive regulator family protein, isocitrate dehydrogenase III, RING-type ubiquitin ligase, rhodanese/cell cycle control phosphatase superfamily protein, and 1-amino-cyclopropane-1-carboxylate synthase 7. The five other proteins did not have interactions with each other. Within the AtGRFs, they did have interactions with each other, except for AtGRF1 and AtGRF6 (Figure 10b). These findings demonstrate the importance of the phosphorylation binding mode of the 14-3-3s and their interactions with their target proteins to ensure their regulatory functions in the organism, and they deepen the comprehension of the functional proteome-wide regulatory network.

### 2.10. Subcellular Localization and Gene Silencing of SlTFT4

**Figure 11:**
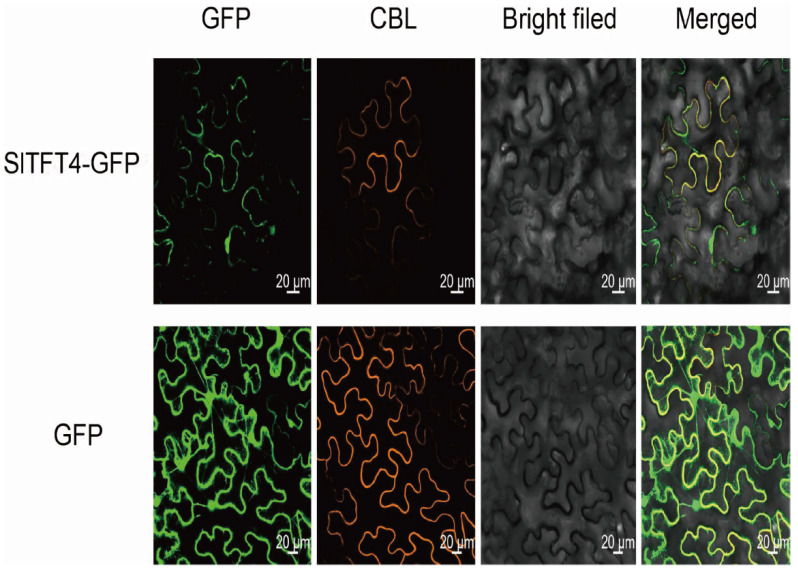
Subcellular localization of SlTFT4. The name of the constructed vector is shown on the left. Images under bright field and fluorescence are displayed. The scale bar is 20 μm.

We performed the subcellular localization of SlTFT4 using tobacco leaves as material to further explore the role of SlTFT4. The results of the localization of the SlTFT4-GFP signal, determined by confocal microscopy, showed that the SlTFT4-GFP fusion protein was detected to emit intense fluorescence in both the cytoplasm and the PM, using the membrane localization marker (CBL) as a reference. Thus, SlTFT4 appears to be co-localized in the cytoplasm and the PM (Figure 11). This is largely consistent with the previous results in which SlTFT4 was predicted to be located mainly in the cytoplasm without a transmembrane helix, indicating the reliability of the electronic predictions and again suggesting the possibility of SlTFT4 as a membrane protein.

VIGS was developed to exploit dsRNA-mediated disease resistance defense mechanisms in plants, and it is one of the most effective reverse genetics techniques used to characterize gene function. The results of our silencing and functional analysis of *SlTFT4* using VIGS technology showed that 14 d after inoculation, *SlTFT4* underwent gene silencing at the post-transcriptional level, with a highly significant 65% decrease in expression compared to the negative control (Supplementary Figure S4a). After 12 h of drought stress and salt stress, all the plants showed some degree of phenotypic changes compared to 0 h. Among them, the *pTRV1-pTRV2-SlTFT4* plants showed more severe yellowing and curling of leaves than the negative control and the positive control plants; in particular, the degree of wilting was more severe under salt stress (Supplementary Figure S4b). 

**Figure 12:**
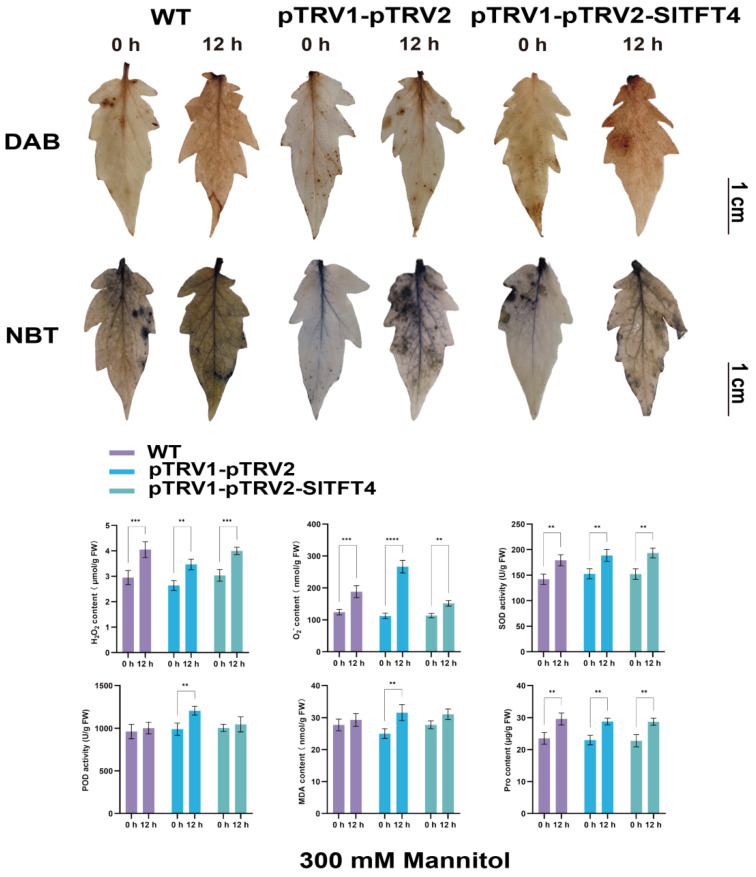
DAB and NBT staining and physiological indices in leaves of different tomato plants under drought stress. WT: untreated wild type plants; pTRV1-pTRV2: control plants injected with an empty vector; pTRV1-pTRV2-SlTFT4: treated plants injected with an *SlTFT4* silencing vector. The upper part of the figure shows the DAB and NBT staining of the leaves under different treatment periods of drought stress. The scale bar of the leaves is 1 cm. The physiological indexes of the leaves under different treatment periods of drought stress are measured below. Error bars are the SD of three independent biological replicates, presented as mean ± SD. The statistical significance of differences was confirmed by Dunnett’s multiple comparison test (** *p* < 0.01, *** *p* < 0.001, and **** *p* < 0.0001).

We further collected the top leaves of plants after drought and salt stress for DAB and NBT staining and measured the enzymatic activities and physiological index contents of their biological antioxidant and reactive oxygen species (ROS) scavenging systems to systematically determine the effect of silencing *SlTFT4* on the drought and salt tolerance of the tomato plant. The results showed that after 12 h of drought stress, the contents of superoxide anion (O_2_^−^), hydrogen peroxide (H_2_O_2_), and proline (Pro) were increased in the leaves of all the plants compared to 0 h. However, the O_2_^−^ content produced by the pTRV1-pTRV2-SlTFT4 plants was less than that of the control by about 20%, and the superoxide dismutase (SOD) activity increased by about 10% without significant differences in the peroxidase (POD) activity and malondialdehyde (MDA) content, implying that their antioxidant defense system functioned relatively normally under drought stress and that the cell membrane did not suffer much damage (Figure 12).

**Figure 13:**
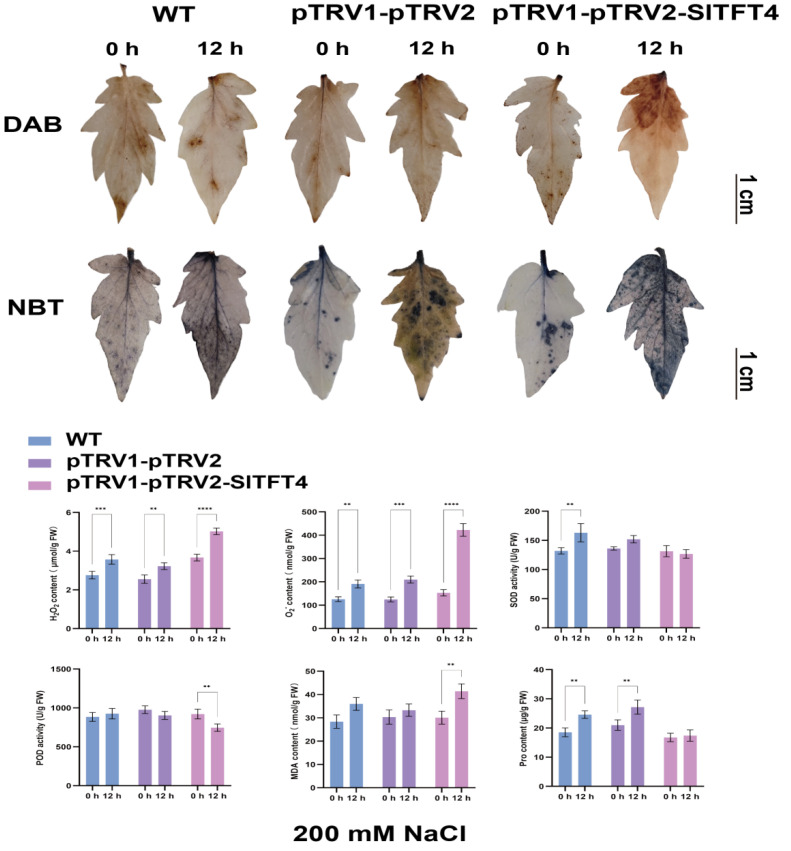
DAB and NBT staining and physiological indices in leaves of different tomato plants under salt stress. WT: untreated wild type plants, pTRV1-pTRV2: control plants injected with an empty vector, pTRV1-pTRV2-SlTFT4: treated plants injected with an *SlTFT4* silencing vector. The upper part of the figure shows the DAB and NBT staining of the leaves under different treatment periods of salt stress. The scale bar of the leaves is 1 cm. The physiological indexes of the leaves under different treatment periods of salt stress are measured below. Error bars are the standard SD of three independent biological replicates, presented as mean ± SD. The statistical significance of differences was confirmed by Dunnett’s multiple comparison test (** *p* < 0.01, *** *p* < 0.001, and **** *p* < 0.0001).

Interestingly, after 12 h of salt stress, although the H_2_O_2_ and O_2_^−^ contents of both the control and the pTRV1-pTRV2-SlTFT4 plants increased with significant differences, the increase in the contents of both was highest in the pTRV1-pTRV2-SlTFT4 plants and both were 30% and 50% higher than the control, respectively. The pTRV1-pTRV2-SlTFT4 plants showed no significant change in SOD activity, and the Pro content did not change significantly, while the POD activity was decreased by about 25% and the MDA content was increased by about 35%, indicating that the antioxidant and the ROS defense systems of the pTRV1-pTRV2-SlTFT4 plants were greatly affected, resulting in their inability to catalyze the timely degradation and enhanced oxidation, which caused more serious damage to the cells (Figure 13). The above phenotypic, physiological, and biochemical measurements suggest that silencing *SlTFT4* decreases the tolerance of tomato plants to salt stress and that it may be a candidate gene involved in the salt stress response.

## 3. Discussion

### 3.1. Significance of the Study of SlTFTs and Its Structural Characterization

It is well known that 14-3-3 proteins are a family of proteins widely expressed in eukaryotes, and different 14-3-3 proteins play different regulatory roles in various physiological processes and tissues of plants. Therefore, it is important to explore the functions of plant 14-3-3 proteins. Due to the deterioration of the environment, abiotic stresses have an increasing impact on plant physiology and crop yield, and the study of plant abiotic stress mechanisms is fundamental to enhancing and improving plant stress tolerance. The 14-3-3 proteins play critical functions in abiotic stresses, and the research targeting them has been an important breakthrough in solving stress problems [57,58].

In the present study, we identified 13 SlTFTs from the tomato genome, and we found one more SlTFT compared with the results of the previous studies [12]. The updated version of the plant genome database, the continuous improvement of the annotation, and more refined gene identification and classification are the most likely reasons for such discrepant results [59,60]. We found that the vast majority of SlTFTs are unstable, with their protein MW mostly around 30 kD, and they are all acidic and hydrophilic proteins, which is generally consistent with the published findings [10]. As with the results of previous studies on *A. thaliana* [4], rice [48], soybean [49], and cotton [50], SlTFTs can be classified into two groups, i.e., the ε group and the non-ε group. They have different structural features of exons and introns, and the intron phase in the ε group is diverse and relatively less conserved than that in the non-ε group, but both contain only the same protein structural domain, suggesting that their main functions may be consistent. However, although the central region of each SlTFT subgroup member is highly conserved, there are variations in the motifs within the N-terminal (affecting the binding to different membranes) and the C-terminal (directly involved in interactions with target proteins) and the structural domains, which may generate functional specificity while forming many heterodimers due to their being the core structures of the 14-3-3 proteins as they perform their different functions [61,62]. Our predicted subcellular localization results indicate that SlTFTs are mainly located in the cytoplasm, with individual distribution in the nucleus, while our transmembrane helix prediction results indicate that the majority of SlTFTs have no transmembrane helix and are probably membrane proteins. For example, SlTFT4 was predicted to be located mainly in the cytoplasm without transmembrane helices. To further confirm the reliability of the electronic prediction results, we conducted molecular experiments to validate the subcellular localization of SlTFT4. The results showed that SlTFT4 co-localized on the cytoplasm and PM. This indicates that the predicted results are still informative, although with some deviations. The previous findings suggest that 14-3-3 proteins are mainly distributed in the cytoplasm but are also present in the PM, nucleus, Golgi apparatus, chloroplasts, and mitochondria [63,64], which also provides indirect support for our findings. In addition, the remaining members of the SlTFTs, except SlTFT8, have the same or similar typical groove space structure, suggesting that they may have similar functions. SlTFTs generally have multiple distinct phosphorylation sites on their AA sequences. It is hypothesized that proteins with different structures and kinase-specific phosphorylation sites determine the functional diversity of SlTFTs.

In the present study, a phylogenetic tree analysis of 14-3-3 proteins among different species was performed to facilitate a better understanding of the evolutionary relationships of the 14-3-3 family between tomatoes and six other species. All the 14-3-3 proteins were divided into two major groups: the ε group and the non-ε group. The clustering of members into the same group in different species implies that they have similar origins and evolutionary relationships. The different groups contain members of 14-3-3s from multiple species; presumably, their origins appeared before the divergence of monocotyledons and dicotyledons. The results of our evolutionary analysis also suggest that certain subbranches in the different groups may have evolved to produce more loss and amplification events, and presumably, the structure and function of 14-3-3s are more susceptible to divergence, suggesting a diversity of gene expression and function in members of this family [58,65].

### 3.2. Evolutionary Characterization of SlTFTs 

Notably, we observed that all 13 SlTFTs showed a non-random distribution pattern on the tomato chromosomes. They were present on only 8 of the 12 chromosomes, with only one and two members on each of the remaining 5 and 2 chromosomes, except for four members on chromosome 4. Interestingly, almost all of the SlTFTs were distributed at the ends of the chromosomes. This layout is a possible reason for the absence of tandem repeat gene pairs. Usually, the source drivers of gene family amplification mainly include segmental duplication, tandem repeats, and transposition events [66]. Segmental duplication plays a vital part in the amplification of the SlTFT family and is the main mechanism leading to the increase in the number of SlTFTs, a result which is identical to that of the GmGF14 family in soybean [49]. The emergence of new genes is inseparable from the generation of replication events, and the diversity of gene functions requires the continuous replenishment of new genes, which can further help plants to resist the stress of adversity in the process of continuous evolution [67].

Generally, the *K*a/*K*s ratio can be used as a measure of selective pressure, which can provide important clues to species selection and evolutionary processes [68]. Our results show that the *K*a/*K*s ratios of the segmental replication gene pairs of *SlTFTs* are all much smaller than 1, and their average value is only 0.09, indicating that SlTFTs have undergone extremely strong purifying selection during evolution, a result that is essentially the same as the evolutionary process of 14-3-3s in other plants, implying that 14-3-3s evolve very slowly at the plant protein level and have a conserved evolutionary mode [49]. This probably plays an essential part in maintaining the long-term stability of the biological structure of SlTFTs.

It has been shown that at least two large-scale replication events have occurred during the evolution of tomatoes. First, chromosome block replication occurred after monocotyledon–dicotyledon differentiation (approximately 170-235 MYA) [69], followed by polyploid replication after *A. thaliana*–tomato division (approximately 90 MYA) [70]. The second scenario is consistent with our analysis of the divergence times of 14-3-3s among the 10 species, with *A. thaliana* and tomato 14-3-3s producing divisions at 90.21 MYA. Previous authors also estimated that another large-scale replication event occurred within the plant kingdom within the last 30 million years [69], which also coincides with the time of the segmental replication of *SlTFTs* and the time of divergence of 14-3-3s in tomato and potato. Considering the similar number of 14-3-3 family members in *A. thaliana*, tomato, and potato, it suggests that there may have been a horizontal doubling of the *AtGRF* family during this period, while no large-scale gene duplication or loss occurred in the *SlTFT* family.

By constructing the synteny profiles separately, we further understood the synteny relationships between *SlTFTs* and other species and among their respective species in *14-3-3s*. Compared with monocotyledons, dicotyledons had more synteny relationships of the *14-3-3s* with each other. Between tomato and potato, the most synteny relationships of 14-3-3s were observed, probably because they belong to the same family of Solanaceae. Notably, no synteny of the *14-3-3s* was found between tomato and wheat, rice, and maize and between melon and rice, suggesting that *14-3-3s* may not be syntenic between monocotyledons and dicotyledons, indirectly indicating the variability and diversity of *14-3-3s* in structure and function. 

### 3.3. Analysis of Cis-Acting Elements of SlTFTs and Correlation with Expression Patterns

It has been shown that in *A. thaliana*, 14-3-3 proteins can interact with photoperiodic regulatory proteins and bind specifically to phototropin 1 (PHOT1) under red and blue light, respectively [71,72]. In *A. thaliana*, 14-3-3 proteins can also regulate IAA concentration and maintain ET levels [73,74]. In tobacco, 14-3-3 proteins can negatively regulate GA expression [75]. In barley, 14-3-3 protein is induced by ABA signaling and is involved in ABA signaling [37]. *Cis*-elements in the promoter region are required for gene regulation and expression. In particular, the *cis*-acting elements are essential for the rapid and accurate recognition of the genes involved in specific functions in processes associated with plant growth and development and resistance to stress [76]. The *cis*-acting elements we screened in the promoter region of the *SlTFTs* were classified into four major classes. In addition, some *SlTFTs* also contain F-boxes with substrate recognition properties during ubiquitin-mediated protein hydrolysis and W-boxes with binding sites for WRKY TFs. The *cis*-acting elements suggest that members of the *SlTFT* family are possibly related to specific growth and development processes, regulated by multiple stress signals and associated with multiple phytohormones responses. 

The results of the correlation analysis between the number of *cis*-acting elements and the expression patterns under different abiotic stresses or phytohormone treatments also showed that *SlTFT4/7/10/11*, whose expression pattern changed significantly under salt and drought stress, had many *cis*-acting elements that responded to salt and drought. *SlTFT4/10/11* had *cis*-acting elements that responded to ABA; so, they showed significant changes in expression patterns upon ABA treatment. *SlTFT7* did not contain a *cis*-acting element in response to MeJA; so, its expression pattern did not change significantly in response to the MeJA treatment. Of course, there was also an exception that *SlTFT10* did not contain a *cis*-acting element in response to MeJA; yet, its expression pattern changed significantly under the MeJA treatment. It also implies the incomplete uniformity between the number of *cis*-acting elements and expression patterns and the complexity of the regulatory mechanism of gene expression.

These inferences are supported by the previous findings on *cis*-acting elements in the promoter regions of the *14-3-3* family members in soybean [49] and mango [53]. It further suggests that *SlTFTs* play a very important role in growth and development and response to adversity and deserve to be studied in depth. 

### 3.4. Unique Transcriptional Profiles and Expression Patterns of SlTFTs

In addition, we found that the *SlTFTs* were expressed in several tissues and that the expression levels varied greatly between the tissues and periods. This presence of tissue-specific expression during specific growth and development is generally consistent with the previously reported expression patterns of the 14-3-3 family members in cottons [50], apples [52], bananas [18], grapes [51], and mangoes [53]. It is speculated that the reason for this phenomenon is related to their possible role as tissue-specific regulators. Furthermore, in our results, the *SlTFTs* were generally expressed at low levels in seeds, flowers, and roots and at high levels in leaves, meristematic tissues, and fruits. It is suggested that during fruit development, certain members differ in protein function and may play specific roles. In grapes, most *VviGRFs* are expressed to a considerable extent in different tissues and organs, but some genes show very high or low expression in certain tissues. For example, *VviGRF12* usually has a low expression in most tissues and organs but not in floral organs such as stamens, buds, flowers, and pollen. *VviGRF15* is expressed at high levels in most tissues and organs except leaves, petals, and pollen. *VviGRFs* may be involved in the regulation of grape development and fruit ripening [51]. In cottons, 31 *GhGRFs* also showed different expression levels in the 15 examined tissues. Nine *GhGRFs* were expressed at relatively low levels in all tissues, while another fifteen *GhGRFs* were highly expressed in all test tissues, indicating that they are involved in the whole process of cotton growth and development. In addition, the expression levels of *GhGRF8-A*/*6-A*/*6-D* in ovules at 0 and 1 d after anthesis were similar to those of these 15 genes, and they may play important roles as tissue-specific regulators [50]. The above findings provide a strong corroboration of our findings. 

Meanwhile, substantial evidence suggests that *14-3-3s* are involved in plant responses to various stresses. The 14-3-3 family members similarly exhibit differential transcript accumulation patterns in response to abiotic stresses, suggesting that they play potentially different roles in regulating abiotic stress responses. For example, in grapes, six *VviGRFs* were significantly up- or down-regulated in their expression in response to cold and heat stresses, suggesting a possible role in abiotic stress responses [51]. In mangoes, drought, salt, and low-temperature stresses affected the expression levels of *Mi14-3-3s*, and different *Mi14-3-3s* responded differently to these stresses [53]. In bananas, *MaGRF2*/*3*/*25* showed similar expression patterns after salt, cold, and osmotic treatments, respectively, indicating that they function similarly in the two cultivars (BX and FJ) under cold, salt, and osmotic treatments. However, six, ten, and six genes exhibited differential expression patterns between BX and FJ under cold, salt, and osmotic stresses, respectively [18]. In tomatoes, *SlTFT1*/*4*/*7*/*10* were significantly up-regulated in young tomato roots under salt stress [12].

We found that the expression pattern of *SlTFTs* in leaves was affected by different abiotic stresses and phytohormone treatments, and certain *SlTFTs* were rapidly induced and persistently highly expressed during specific periods, suggesting that they have specific roles under different stresses and phytohormone treatments. *SlTFT4*/*10* can simultaneously respond to all processing conditions in this study, and they may be key genes in the *SlTFT* family, involved in defense and response to external stress. In addition, we also noticed that the expression of *SlTFTs* tended to be more responsive to drought stress and ABA treatment compared to the rest of the treatments. It has been shown that under drought stress, for the tomato ABA-deficient mutant *notabilis*, inoculation with AM significantly up-regulated the expression of *SlTFT5*/*7*/*9*/*10* to maintain WUE and improve drought tolerance [42]. In conclusion, all the above reports provide indirect support for our findings.

### 3.5. The Constructed Protein Network That Interacts with SlTFTs Deepens the Knowledge

Generally, 14-3-3 proteins are required to respond to adversity stress by combining with target proteins. As 14-3-3 proteins require a low specificity of recognition sequences and diverse recognition modes in recognizing target proteins, the types of target proteins regulated by 14-3-3 proteins are abundant [31,77]. Our constructed interaction network between SlTFTs and the other proteins showed that all 13 SlTFTs interacted with five (SlBSL3, SlPP2C, SlBSU1, SlCDC25, and SlBSL1) other proteins belonging to dual-specificity tyrosine-phosphatase, serine/threonine-protein phosphatase, and protein kinase A-like kinase. This is consistent with the study that 14-3-3 proteins mainly combine with their interacting target proteins through phosphorylation, which in turn regulates the function of the target protein, and their structural requirement for the interacting target protein is to contain a phosphorylated Ser or Thr sequence [77,78]. Interestingly, there are fewer interactions within the SlTFTs and more interactions in the five other proteins. It is hypothesized that they exert their regulatory effects mainly by binding to phosphorylated target proteins rather than by their phosphorylation. 

Subsequently, we also constructed the interaction network between AtGRFs and other proteins, and we observed that all 14 AtGRFs interacted with five (AtBZR1, AtIDH-III, AtCNI1, AtCDC25, and AtACS7) other proteins belonging to the brassinosteroid signaling positive regulator family protein, isocitrate dehydrogenase III, RING-type ubiquitin ligase, the rhodanese/cell cycle control phosphatase superfamily protein, and 1-amino-cyclopropane-1-carboxylate synthase 7. In particular, there were more interactions within the AtGRFs, while there were no interactions among the five other proteins. Although 14-3-3 proteins mainly exert their regulatory effects by interacting with target proteins, the interactions between the different internal members may enhance their regulatory effects in some physiological processes [31,58]. As different family members of 14-3-3 proteins have their specific regulatory functions in rice, for example, OsGF14b is associated with drought resistance [79], OsGF14c is considered as a negative regulator of flowering [80], and OsGF14e negatively regulates cell death and the disease resistance response [81]. In maize, ZmGF14-4 specifically binds to disease and resistance-related proteins during endosperm development, while ZmGF14-6 mainly interacts with metabolic and cytoarchitecture-related proteins [82]. Therefore, our findings corroborate the importance of the phosphorylation binding mode of 14-3-3 proteins and their interactions with their target proteins to ensure their regulatory functions in organisms, and they deepen the understanding of the functional proteome-wide regulatory network. They also lay the groundwork for further investigations into the regulatory mechanism of phosphorylation and phytohormones signaling pathways in 14-3-3 proteins and their roles in plant development and the response to adversity stress.

### 3.6. Functional Analysis Revealed That SlTFT4 Is a Candidate Gene for Salt Tolerance

Typically, the consideration of the overexpression of *14-3-3s* in transgenic plants has been used to elucidate the contribution of the corresponding proteins in stress resistance [43,44]. For example, the overexpression of *AtGRF9*, *At14-3-3λ*, and *ZmGF14-6*/*OsGF14c* improved drought resistance in transgenic *A. thaliana*, cotton, and rice, respectively [19,20,21,22], while the silencing of *AtGF14m* improved drought resistance in *A. thaliana* at the seed germination and seedling stages [23]. The At14-3-3λ/κ have been shown to negatively regulate salt tolerance [20,24]. The transgenic *A. thaliana* overexpressing *SlTFT6* and *SlTFT7*, respectively, both showed great growth promotion under LP stress [44]. Moreover, the transgenic *A. thaliana* overexpressing *SlTFT4* showed significantly enhanced growth under alkaline stress [43]. In the present study, we found that silencing *SlTFT4* significantly increased the content of H_2_O_2_, O_2_^−^, and MDA in leaves, while significantly decreasing POD activities, which caused a greater impact on the biological antioxidant and ROS defense systems, increased the oxidation, thereby increasing the degree of cellular damage, and led to an increase in leaf wilting, reducing the tolerance of tomato plants to salt stress. Therefore, *SlTFT4*, identified by VIGS technology, can be used as a candidate gene for subsequent studies to improve the understanding of the salt tolerance function studies on tomatoes.

## 4. Materials and Methods

### 4.1. Identification and Characterization of SlTFT Family

The genome-wide data were downloaded from the International Tomato Annotation Group website (https://www.sgn.cornell.edu/organism/Solanum_lycopersicum/genome, accessed on 23 January 2022), and the HMM profile (PF00244), downloaded from the protein families database (http://pfam.xfam.org/, accessed on 23 January 2022), was loaded into the HMMER search (https://www.ebi.ac.uk/Tools/hmmer/, accessed on 23 January 2022) at the default inclusion threshold. The potential SlTFTs identified in the genomic database were retrieved by the above steps (Supplementary Table S5). All predicted protein sequences of SlTFTs were uploaded to the Simple Modular Architecture Research Tool database (http://smart.embl-heidelberg.de/, accessed on 24 January 2022) and HMMER to verify the 14-3-3 domain presence. Subsequently, sequences that did not contain the 14-3-3 domain (Solyc02g086840.3.1, Solyc08g008455.1.1, Solyc09g065270.3.1, and Solyc03g114320.3.1) were artificially discarded (Supplementary Table S6). The physicochemical parameters of the SlTFTs were measured using the ExPASy server tool (http://web.expasy.org/protparam/, accessed on 24 January 2022). In addition, the SubCELlular LOcalization predictor website (http://cello.life.nctu.edu.tw/, accessed on 25 January 2022) was used to forecast the possible subcellular localization results of the SlTFTs. The tertiary structure of the SlTFTs was forecasted using the SWISS-MODEL server (https://swissmodel.expasy.org/interactive, accessed on 25 January 2022). The identification of possible phosphorylation sites of the SlTFTs was conducted using NetPhos 3.1 server (https://services.healthtech.dtu.dk/service.php?NetPhos-3.1, accessed on 26 January 2022). The prediction of the transmembrane helices of the SlTFTs was performed using the TMHMM online software tool (https://services.healthtech.dtu.dk/service.php?TMHMM-2.0, accessed on 29 January 2022). The details of the bioinformatics analysis are as follows: after opening the homepage of the above website, click on the corresponding input box or analysis button, paste the AA sequence of each SlTFT for analysis, and then obtain bioinformatics information, such as various physicochemical properties, subcellular localization, tertiary structure, possible phosphorylation sites, and transmembrane helices of the protein.

### 4.2. Phylogenetic Tree, Gene Structure, Conserved Domains, and Motif Analysis of SlTFTs

The protein sequences of SlTFTs were used to create a phylogenetic tree, and the CDS and genomic base sequences of *SlTFTs* were used to analyze the gene structure. The conserved domains and motifs of SlTFTs were evaluated using the HMMER search and the Multiple Expectation Maximization for Motif Elicitation website (http://meme-suite./org/tools/meme, accessed on 27 January 2022), respectively. Lastly, the above data were visualized using TBtools [83].

### 4.3. Phylogenetic Tree Analysis of SlTFTs among Different Species

To explore the phylogenetic relationships among SlTFT families in different species, we first obtained the 14-3-3 protein sequences of six other species using HMMER search screening. Then, the 14-3-3 protein sequences of all seven species were imported into MEGA 11 software, and the phylogenetic tree analysis was performed by the maximum likelihood method with bootstrap analysis of 1000 replicates.

### 4.4. Chromosome Distribution, Replication Events, and Synteny Analysis of SlTFTs

The distribution of *SlTFTs* on chromosomes was calculated and plotted using TBtools. Gene replication events were calculated and plotted for *SlTFTs* using the MCScanX toolkit [84]. The ratio of *K*a and *K*s in duplicated gene pairs in *SlTFTs* was calculated by the KaKs_Calculator 3.0 software [68]. The replication date in each gene pair was evaluated using the equation T = *K*s/2λ, where λ was assumed to be 1.5 × 10^−8^ synonymous/substitution site/year [69]. Single-copy homologous genes were obtained using diamond comparison in the OrthoFinder 2.5.2 software [85], and evolutionary divergence trees of different species were constructed using the RAxML 8.0 tool [86], and fossil times were added to the evolutionary trees using the TimeTree database (http://www.timetree.org/, accessed on 7 February 2022). The fossil calibration times were 40.3 to 51.9 MYA for rice, maize, and wheat, and 109.0 to 123.5 MYA for *A. thaliana*, grapes, and poplars. Then, the mcmctree command in the PAML 4.9 software [87] was used to estimate the divergence times of different species. In addition, the synteny of *14-3-3s* between different species was calculated, analyzed, and visualized using the dual synteny plotter module in TBtools.

### 4.5. Analysis of Cis-Acting Elements in the Promoter Region of SlTFTs

The sequences within 2000 base pairs upstream of each *SlTFT* initiation codon were extracted from the tomato genome database and subsequently collated and submitted to the PlantCARE database (http://bioinformatics.psb.ugent.be/webtools/plantcate/html, accessed on 8 February 2022) for *cis*-acting element screening and identification. Visualization was performed using TBtools.

### 4.6. Transcriptional Profiling of SlTFTs

The transcriptional profiling data of *SlTFTs* in different tissues, such as seeds, roots, meristematic tissues, leaves, flowers, and fruits, were analyzed using the TomExpress database (http://tomexpress.toulouse.inra.fr/, accessed on 10 February 2022), a unified tomato RNA-Seq platform. Cluster heat maps were drawn using the OmicShare tool (https://www.omicshare.com/tools, accessed on 10 February 2022), a free online data analysis platform.

### 4.7. Plant Material and Treatments

Seeds (*Solanum lycopersicum* var. M82 is a representative material of the processing of tomato, which is collected and preserved by Horticultural Crops Research, Xinjiang Academy of Agricultural Sciences) were planted in potting soil (1:3 ratio of vermiculite to nutrient soil) and grown in an artificial climate chamber at the appropriate temperature and humidity, with the light and dark cycles set to 16 h/8 h. After 4 weeks, seedlings of uniform growth were watered with 300 mM D-mannitol (CM7091-1kg, Coolaber, Beijing, China) and 200 mM NaCl (CS9971-1kg, Coolaber, Beijing, China) solutions to simulate drought and salt stress responses, respectively, and seedlings without any treatment under the above standard degree conditions were used as controls. The leaves of seedlings with essentially the same growth were uniformly sprayed with 100 μM ABA (PH109X-2×1 mL, Coolaber, Beijing, China) and 100 μM MeJA (CJ6691-5 mL, Coolaber, Beijing, China) solutions, respectively, for phytohormone treatment response analysis, with leaves of seedlings sprayed with ddH_2_O as controls. In each of the above treatments, the top first fully expanded leaves of three plants were mixed and harvested as one biological replicate and three technical replicates, and all the abiotic stress and phytohormone experimental samples were collected and obtained at 0 h, 3 h, 12 h, and 24 h post-treatment, respectively. The samples were first rapidly frozen in liquid nitrogen and subsequently stored in an ultra-low temperature refrigerator at −80 °C until they were used.

### 4.8. Total RNA Extraction, Quantitative Real-Time PCR (qRT-PCR), and Statistical Analysis

Total RNA was extracted from the lyophilized samples using the Polysaccharide Polyphenol Plant Total RNA Extraction Kit (DP441, Tiangen, Beijing, China) and processed with RNase-free DNAase following the manufacturer’s protocol. Total RNA was reverse transcribed into cDNA using 5 × All-In-One RT MasterMix (with AccuRT Genomic DNA Removal Kit) (G492, ABM, Vancouver, BC, Canada). qRT-PCR was performed on a LightCycler^®®^ real-time fluorescent quantitative PCR system (Roche, Basel, Switzerland) using ChamQ Universal SYBR qPCR Master Mix (Q711, Vazyme, Nanjing, China). The amplification process was pre-denaturation: 95 °C, 30 s, one cycle; followed by denaturation: 95 °C, 5 s; annealing extension: 60 °C, 30 s, 40 cycles; and finally a melting curve was obtained: 95 °C, 15 s, 60 °C, 60 s, 95 °C, 15 s, one cycle. The specific primers and the internal reference sequence of *Actin* (*Solyc03g078400.2*) used in this study for *SlTFTs* were designed using NCBI Primer-BLAST (https://www.ncbi.nlm.nih.gov/tools/primer-blast/index.cgi?LINK_LOC=BlastHome, accessed on 13 February 2022) and synthesized by Sangon Biotech (Shanghai, China) Co., Ltd., and then, the amplification validity study was performed first using RT-PCR, and primers with unique specific bands for the target fragment (80–150 bp range) were selected for subsequent qRT-PCR assay analysis, and the details are shown in Supplementary Table S7. All qRT-PCR assays were performed three times as independent biological replicates, and the mean of the cycle threshold (C_t_) was obtained. Relative gene expression levels were calculated using the 2^−ΔΔCt^ method [88]. Three independent biological replicates were averaged and analyzed for each assay. One-way analysis of variance was performed using GraphPad Prism for Windows (version 9.0.0, GraphPad Software, San Diego, CA, USA), followed by Dunnett’s multiple comparison test.

### 4.9. Protein Interaction Network Analysis

Protein interactions were queried according to String 11.5 (https://cn.string-db.org/, accessed on 15 February 2022). In the basic settings, select the full STRING network (the edges indicate both functional and physical protein associations) as the network type. The line color indicates the type of interaction evidence chosen to indicate the meaning of the network edges. Active interaction sources include text mining, experiments, databases, co-expression, neighborhood, gene fusion, and co-occurrence. The minimum required interaction score is medium confidence (0. 40). The network display mode in the advanced settings is set to interactive SVG (the network is a scalable vector graphic [SVG]; interactive). Select hide disconnected nodes in the network in the network display option. Thereafter, the interaction network of SlTFTs with other proteins was visualized using Cytoscape 3.9.1 (https://cytoscape.org//, accessed on 15 February 2022).

### 4.10. Subcellular Localization

Briefly, the method of subcellular localization adopted in the present study comprised the following. The CDS of *SlTFT4* was amplified using cDNA as a template by the upstream primer 5′- GCGTCGACATGGCTGACTCTTCGCGTGA-3′ (*Sal* I enzyme cut site) and the downstream primer 5′-GCTCTCTAGACTGCTGCCGCCTCGCCTGAC-3′ (*Xba* I enzyme cut site), respectively. After correct sequencing, the agarose gel recovery product of the target fragment was ligated into the pCAMBIA super1300-GFP plant expression vector, which was also double-cleaved with *Sal* I and *Xba* I, and after correct sequencing again, the pCAMBIA super1300-SlTFT4-GFP vector was constructed. The pCAMBIA super1300-GFP empty was used as a control, and the CBL was used as a reference. The vector plasmid was transformed into *Agrobacterium tumefaciens* GV3101, and after overnight incubation, it was collected by centrifugation and suspended using the suspension, and the concentration was adjusted to OD_600_ = 0.8. They were then mixed separately at a 1:1 (*v*/*v*) ratio and incubated at room temperature for 3 h in the dark before infiltration from the abaxial side of *Nicotiana benthamiana* leaves using a needleless 1 mL syringe, and the infiltrated plants were incubated in the dark for 12 h and then grown for an additional 5 d under a 16 h/8 h light/dark cycle. Lastly, confocal laser scanning microcopy (LSM 800, Zeiss, Germany) was used for observation and photography.

### 4.11. VIGS Vector Construction and the Process of Gene Silencing and Physiological Indices Determination

The VIGS vector for the target gene was designed and constructed using the SGN VIGS tool (https://vigs.solgenomics.net/, accessed on 17 February 2022). The pTRV2 recombinant vector was transformed into *A. tumefaciens* GV3101 (pSoup-p19). *A. tumefaciens* containing pTRV1, pTRV2 empty, and pTRV2 recombinant vectors were incubated in a rotary shaker (28 °C, 200 rpm). The cells were collected by centrifugation at 4000 rpm for 10 min and resuspended in appropriate volumes of resuspension solution (10 mM MgCl_2_, 10 mM MES, and 150 μM acetosyringone) to a final concentration of OD_600_ = 1.0. The pTRV1-pTRV2 empty and pTRV1-pTRV2 recombinant vector resuspensions were mixed at a ratio of 1:1 (*v*/*v*) and incubated for 3 h at 28 °C, protected from light, and then infiltrated into the abaxial surface of the leaves of 4-week-old tomato plants using a de-needled 1 mL syringe. After 12 h of dark treatment, a normal 16 h/8 h photoperiod was performed. Plants without treatment were used as negative controls, and plants inoculated with pTRV1-pTRV2 empty were used as positive controls. After 14 d, salt and drought stresses were applied as described previously, respectively, and the top leaves were collected from the same parts at 0 h and 12 h post-stress and stained using DAB solution (1 mg/mL, pH 3.8) (SL1805, Coolaber, Beijing, China) and NBT solution (0.1%, pH 7.8) (SL18061, Coolaber, Beijing, China), respectively. According to the manufacturer’s instructions, six test kits (SA-1-G, H2O2-1-Y, MDA-1-Y, PRO-1-Y, SOD-1-Y, and POD-1-Y, Comin, Suzhou, China) were used sequentially to detect O_2_^−^, H_2_O_2_, MDA, Pro, SOD, and POD activity/content, and three replicates were performed.

## 5. Conclusions

In summary, this research provides a comprehensive and systematic description of the SlTFT family. We found 13 SlTFTs divided into two groups. The *SlTFTs* were non-randomly distributed on chromosomes, and the promoter regions contained more *cis*-acting elements involved in abiotic stress tolerance and phytohormone responses. Gene expression pattern analysis indicated specific roles for certain *SlTFTs* under different abiotic stresses and phytohormone treatments. *SlTFT4* may be a candidate gene involved in the salt stress response. Our results provide comprehensive information on the characteristic structure, evolutionary history, expression patterns, and protein interactions of the SlTFT family, which is a significant guide for the further elucidation of this family. It also lays the foundation for the subsequent in-depth study of the molecular and biological functions of this family and the screening of family members for future application in variety improvement.

## Figures and Tables

**Table 1:** Identification and characterization of SlTFTs.

Group	Name	ID	Length (AA)	MW (Da)	pI	GRAVY	Instability Index	Subcellular Localization
non-ε	SlTFT1	Solyc11g010470.2.1	249	28,201.16	4.76	−0.0261	37.97 (Stable)	Cytoplasmic (2.968)
non-ε	SlTFT2	Solyc12g057120.2.1	401	45,149.05	4.67	−0.689	54.17 (Unstable)	Cytoplasmic (2.695)
non-ε	SlTFT3	Solyc04g074510.3.1	268	30,439.91	5.4	−0.353	42.24 (Unstable)	Cytoplasmic (2.492)
non-ε	SlTFT4	Solyc02g063070.3.1	260	29,338.81	4.66	−0.517	43.74 (Unstable)	Cytoplasmic (3.355)
non-ε	SlTFT5	Solyc04g012120.3.1	255	28,795.31	4.74	−0.525	41.08 (Unstable)	Cytoplasmic (3.528)
non-ε	SlTFT6	Solyc11g010200.2.1	259	29,107.68	4.76	−0.494	39.24 (Stable)	Cytoplasmic (3.666)
ε	SlTFT7	Solyc04g074230.3.1	243	27,757.46	5.31	−0.344	52.12 (Unstable)	Nuclear (1.160)
ε	SlTFT8	Solyc12g010860.2.1	261	29,478.94	4.61	−0.531	43.40 (Unstable)	Cytoplasmic (2.519)
ε	SlTFT9	Solyc07g053260.3.1	261	29,431.93	4.74	−0.538	47.55 (Unstable)	Cytoplasmic (2.867)
non-ε	SlTFT10	Solyc04g076060.3.1	295	33,701.28	5.14	−0.397	40.59 (Unstable)	Cytoplasmic (2.356)
non-ε	SlTFT11	Solyc03g034180.3.1	258	29,122.59	4.69	−0.492	44.61 (Unstable)	Cytoplasmic (3.792)
ε	SlTFT12	Solyc05g012420.3.1	367	42,073.87	5.33	−0.387	50.02 (Unstable)	Cytoplasmic (1.076)
ε	SlTFT13	Solyc01g010360.3.1	249	28,193.67	4.95	−0.718	51.66 (Unstable)	Cytoplasmic (2.979)

Length (AA): the length of the protein in AA; MW (Da): molecular weight in Da; pI: isoelectric point; GRAVY: grand average of hydropathy.

## Data Availability

Not applicable.

## Supplementary Materials

The following supporting information can be downloaded at: https://www.mdpi.com/article/10.3390/plants11243491/s1, Figure S1: Synteny maps between *SlTFTs* and *14-3-3s* of other four species, respectively. The different colored rectangles represent the chromosomes of different species. The gray lines indicate synteny between tomato genome and other species. The red line indicates the synteny between *SlTFTs* and *14-3-3s* of other species; Figure S2: Expression patterns of SlTFTs under normal conditions (control for stress treatments). A to M represent *SlTFT1* to *SlTFT13*, respectively. The horizontal coordinates in the figure are the four different treatment periods, and the vertical coordinates are the relative expression of each SlTFT gene. Error bars are standard deviations (SD) of three independent biological replicates, presented as mean ± SD. Statistical significance of differences was confirmed by Dunnett's multiple comparison test (* *p* < 0.05, ** *p* < 0.01, *** *p* < 0.001, and **** *p* < 0.0001); Figure S3: Expression patterns of *SlTFTs* in response to ddH2O treatment (control for phytohormone treatments). A to M represent *SlTFT1* to *SlTFT13*, respectively. The horizontal coordinates in the figure are the four different treatment periods, and the vertical coordinates are the relative expression of each *SlTFT* gene. Error bars are SD of three independent biological replicates, presented as mean ± SD. Statistical significance of differences was confirmed by Dunnett's multiple comparison test (* *p* < 0.05, ** *p* < 0.01, *** *p* < 0.001, and **** *p* < 0.0001); Figure S4: Relative expression of *SlTFT4* after silencing and phenotypic changes of tomato plants under stress conditions. (a) qRT-PCR to verify the relative expression of *SlTFT4* after silencing. The horizontal coordinates in the figure represent the different plant types. WT: untreated wild type plants, pTRV1-pTRV2: control plants injected with a empty vector, pTRV1-pTRV2-SlTFT4: treated plants injected with a *SlTFT4* silencing vector. The vertical coordinates represent the relative expression of *SlTFT4* in different plant types. Error bars are SD of three independent biological replicates, presented as mean ± SD. Statistical significance of differences was confirmed by Dunnett's multiple comparison test (* *p* < 0.05, ** *p* < 0.01, *** *p* < 0.001, and **** *p* < 0.0001). (b) Phenotypic changes in different categories of tomato plants under drought and salt stress conditions. The figure represents different plant types and the same treatment period horizontally, and the same plant types and different treatment periods vertically. The upper two columns show the phenotypic changes of different plant types under 300 mM D-mannitol simulated drought stress treatment at 0 h and 12 h treatment periods. The following two columns show the phenotypic changes of different plant types under 200 mM NaCl simulated salt stress treatment at 0 h and 12 h treatment periods. The scale bar of the plants is 5 cm.; Table S1: Sequence information of the 13 identified SlTFTs; Table S2: Results of the TMHMM in this study; Table S3: The list of 7 pairs of fragment duplication events, *Ka*/*Ks* ratios, and their replication date for *SlTFTs*; Table S4: Number of *cis*-acting elements involved in the response to different abiotic stresses and phytohormone treatments; Table S5: Results of the HMMER search in this study; Table S6: Results of verifying the presence of 14-3-3 domain in SMART and HMMER; Table S7: The primer sequences used in this study for qRT-PCR.
