# Supplementary material for: Genome-Wide Identification and Expression Analysis of the 14-3-3 (TFT) Gene Family in Tomato, and the Role of SlTFT4 in Salt Stress
Source: Plants (Basel). 2022 Dec 13;11(24):3491. doi: 10.3390/plants11243491 (PMC9781835; doi:10.3390/plants11243491)
Supplement: Supplementary file 1 [file plants-11-03491-s001.zip › Supplementary Figure S1-S4.pdf]

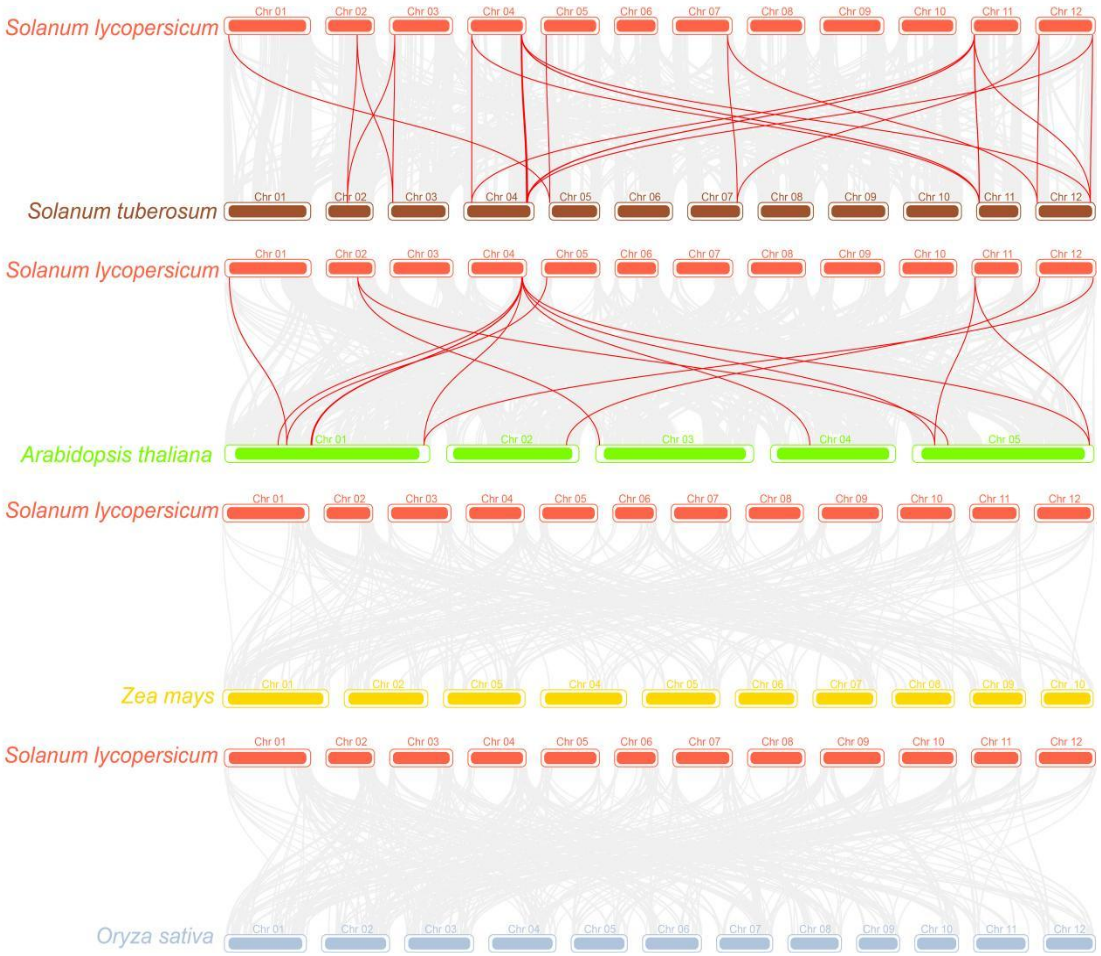

Figure S1. Synteny maps between *SlTFTs* and *14-3-3s* of other four species, respectively. The different colored rectangles represent the chromosomes of different species. The gray lines indicate synteny between tomato genome and other species. The red line indicates the synteny between *SlTFTs* and *14-3-3s* of other species.

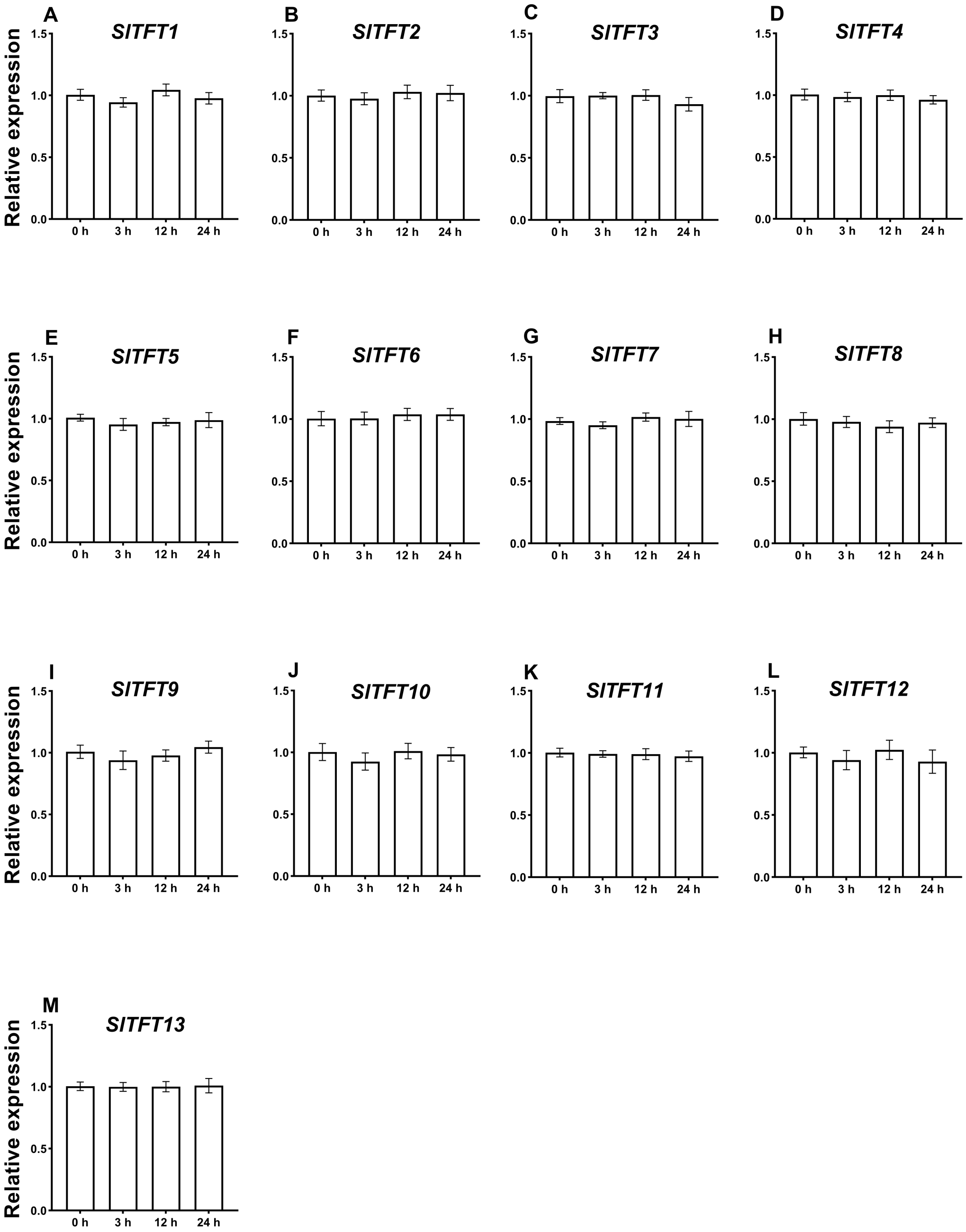

Figure S2. Expression patterns of *SlTFTs* under normal conditions (control for stress treatments). A to M represent *SlTFT1* to *SlTFT13*, respectively. The horizontal coordinates in the figure are the four different treatment periods, and the vertical coordinates are the relative expression of each *SlTFT* gene. Error bars are standard deviations (SD) of three independent biological replicates, presented as mean ± SD. Statistical significance of differences was confirmed by Dunnett's multiple comparison test ($^{*}P$<0.05, $^{**}P$<0.01, $^{***}P$<0.001, and $^{****}P$<0.0001).

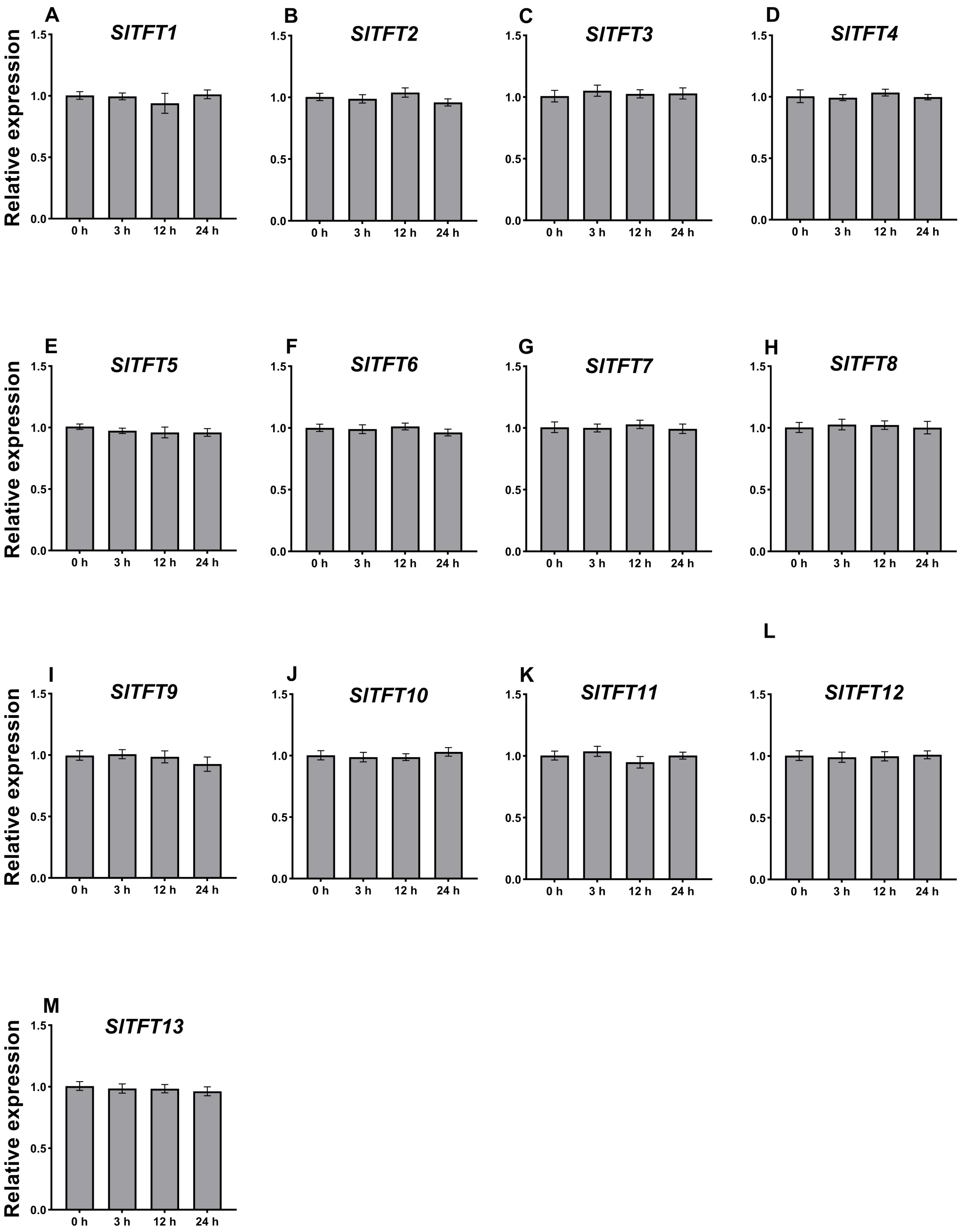

Figure S3. Expression patterns of *SlTFTs* in response to ddH_2O treatment (control for phytohormone treatments). A to M represent *SlTFT1* to *SlTFT13*, respectively. The horizontal coordinates in the figure are the four different treatment periods, and the vertical coordinates are the relative expression of each *SlTFT* gene. Error bars are SD of three independent biological replicates, presented as mean ± SD. Statistical significance of differences was confirmed by Dunnett's multiple comparison test ($^{*}P<0.05$, $^{**}P<0.01$, $^{***}P<0.001$, and $^{****}P<0.0001$).

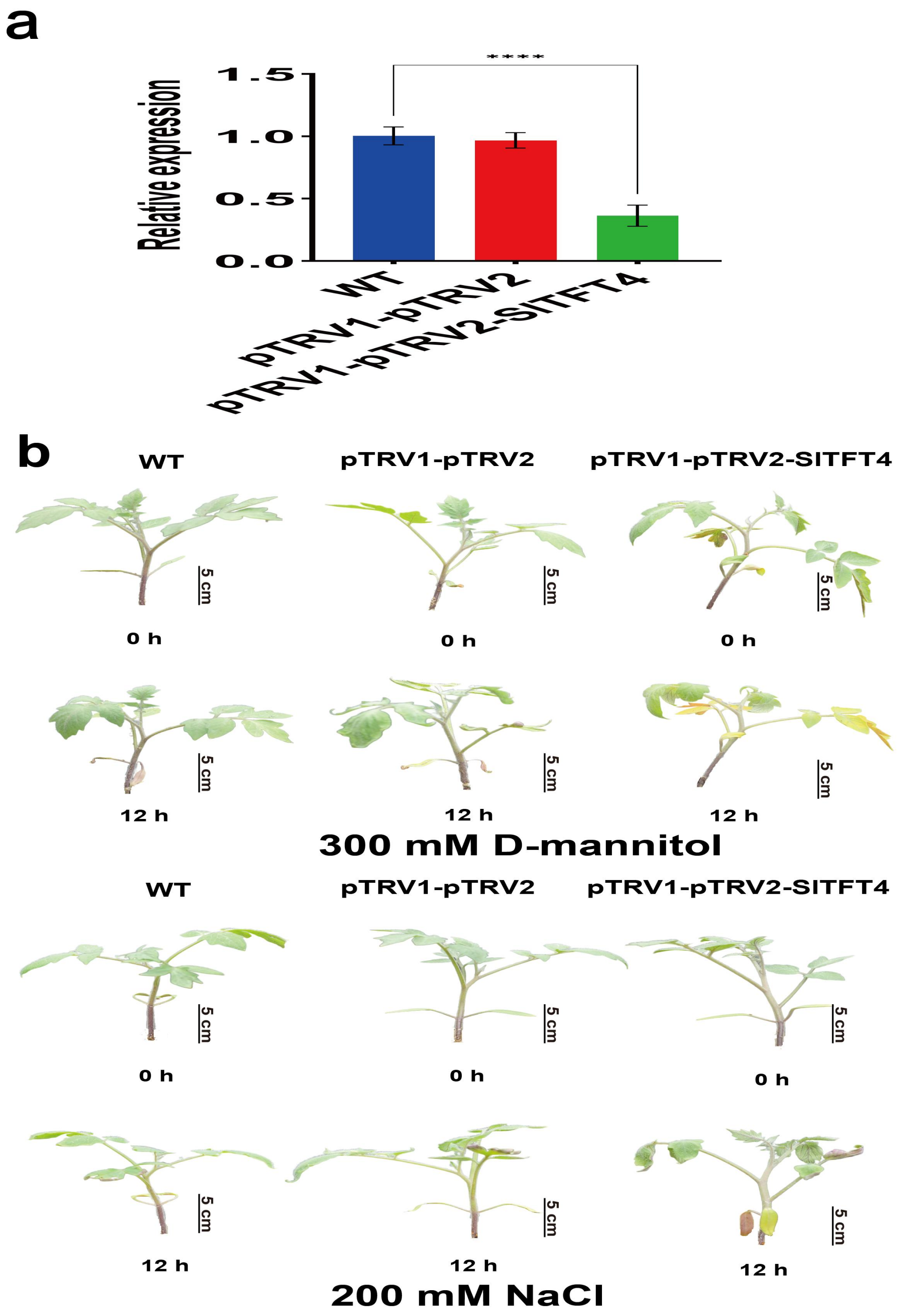

Figure S4. Relative expression of *SlTFT4* after silencing and phenotypic changes of tomato plants under stress conditions. **(a)** qRT-PCR to verify the relative expression of *SlTFT4* after silencing. The horizontal coordinates in the figure represent the different plant types. WT: untreated wild type plants, pTRV1-pTRV2: control plants injected with a empty vector, pTRV1-pTRV2-SlTFT4: treated plants injected with a *SlTFT4* silencing vector. The vertical coordinates represent the relative expression of *SlTFT4* in different plant types. Error bars are SD of three independent biological replicates, presented as mean ± SD. Statistical significance

of differences was confirmed by Dunnett's multiple comparison test ($^{*}P<0.05$, $^{**}P<0.01$, $^{***}P<0.001$, and $^{****}P<0.0001$). **(b)** Phenotypic changes in different categories of tomato plants under drought and salt stress conditions. The figure represents different plant types and the same treatment period horizontally, and the same plant types and different treatment periods vertically. The upper two columns show the phenotypic changes of different plant types under 300 mM D-mannitol simulated drought stress treatment at 0 h and 12 h treatment periods. The following two columns show the phenotypic changes of different plant types under 200 mM NaCl simulated salt stress treatment at 0 h and 12 h treatment periods. The scale bar of the plants is 5 cm.
